# A P3′ methionine-to-phenylalanine substitution in the NSP6–7 substrate peptide alters SARS-CoV-2 M^pro^ dynamics and enhances its processing in living cells

**DOI:** 10.1016/j.jbc.2026.113495

**Published:** 2026-08-26

**Authors:** Asma Fatima, Anupriya M. Geethakumari, Asfia Sultana, S.M. Nasir Uddin, Zeyaul Islam, Prasanna R. Kolatkar, Kabir H. Biswas

**Affiliations:** 1 College of Health & Life Sciences, Hamad Bin Khalifa University, Doha, Qatar; 2 Diabetes Research Center, Qatar Biomedical Research Institute, Hamad Bin Khalifa University, Doha, Qatar

**Keywords:** COVID-19, main protease (M^pro^), molecular dynamics (MD) simulation, peptide substrate, proteolytic cleavage, SARS-CoV-2

## Abstract

The SARS-CoV-2 main protease (M^pro^) cleaves viral polyproteins at 11 sites to release nonstructural proteins required for replication, with site-specific sequence differences influencing processing efficiency. Among these substrates, NSP6–7 is consistently the slowest cleaved, while NSP4–5 is among the fastest. However, the mechanistic basis for slow NSP6–7 processing remains unclear. Here, we combined molecular dynamics (MD) simulations with DuProSense biosensor-based live-cell and *in vitro* assays to show that the P3′ M is a key regulator of NSP6–7 binding, and cleavage by M^pro^ and M-to-F substitution increases its cleavage in living cells. MD simulations of dimeric M^pro^ bound to either NSP4–5 or NSP6–7 revealed increased flexibility and altered positioning of the C-terminal P2′, P3′, and P4′ residues of NSP6–7 compared with NSP4–5. Guided by these observations, we individually replaced these NSP6–7 C-terminal residues with the corresponding residues from NSP4–5. BRET-based cleavage assays using DuProSense biosensor showed that the P3′ M-to-F substitution, but not the P2′ K-to-G or P4′ S-to-R substitutions, increased NSP6–7 cleavage by M^pro^ in living cells, consistent with increased apparent substrate affinity *in vitro*. Comparison of structural dynamics of M^pro^ bound to NSP6–7(M7F) with NSP4–5 and NSP6–7 showed that the P3′ substitution resulted in a dynamic profile more similar to NSP4–5 while introducing distinct NSP6–7(M7F)-specific effects, including enhanced correlated motions and reduced flexibility within C44–L50 residues in M^pro^. Taken together, these results suggest that M7F mutation-induced enhanced cleavage of NSP6–7 arises from both restored and mutation-specific dynamic effects.

The SARS-CoV-2 main protease (M^pro^), also referred to as the 3C-like protease (3CLpro), is a cysteine protease that is essential for viral replication due to its proteolytic processing activity of viral polyproteins into functional nonstructural proteins (1, 2, 3, 4, 5, 6). The structure of M^pro^ comprises three distinct domains, namely, domains I (residues 8–101) and II (residues 102–184), which adopt a chymotrypsin-like fold containing the catalytic dyad residues H41 and C145 (7, 8, 9), and domain III (residues 201–303), which is predominantly α-helical and mediates protease dimerization and is required for enzymatic activity (8, 9, 10). Due to its indispensable role in the viral infection cycle and the lack of closely related human homologs, M^pro^ represents a prominent target for the development of anti-COVID therapeutics (10, 11, 12, 13, 14). Specifically, SARS-CoV-2 M^pro^ cleaves the viral polyproteins pp1a and pp1ab at 11 specific cleavage sites, henceforth referred to as substrate peptides, to free the nonstructural proteins (NSPs) required for viral replication and assembly (1, 2, 3, 4, 5, 6). Importantly, these M^pro^ substrate peptides have distinct amino acid sequences that can influence their binding to and proteolysis by M^pro^ (1, 15, 16, 17, 18, 19, 20, 21, 22, 23, 24). Such variations in cleavage efficiency are believed to determine the order in which polyproteins are processed and the timing of NSP maturation during the viral life cycle (10, 23, 25, 26). Previous studies reporting the cleavage kinetics of M^pro^ substrate peptides in live cells and *in vitro* revealed substrate peptide-dependent differences in proteolytic efficiency, underscoring the influence of substrate peptide sequence on viral polyprotein processing (15, 16, 27). Notably, the NSP6–7 substrate peptide (hereafter referred to as NSP6–7) showed the slowest cleavage kinetics in both live cells and *in vitro*. This observation prompted us to have a detailed investigation into the interaction of NSP6–7 with M^pro^ catalytic site to uncover structural or biochemical determinants underlying its reduced cleavage rate.

NSP6–7 is located between the C-terminal region of NSP6 and the N-terminal region of NSP7. NSP6 is a transmembrane protein that plays a key role in double-membrane vesicle (DMV) formation by organizing and positioning DMV clusters and mediating their interaction with the endoplasmic reticulum (ER) (28). Previous literature has shown the importance of cleavage of NSP6–7 to release NSP6 for its ER zippering activity, which facilitates DMV production (28, 29). The intact NSP6–7 protein intermediate localizes to the Golgi complex and fails to induce ER zippering, suggesting that delayed or incomplete cleavage at this site may regulate DMV biogenesis. Interestingly, a variant of NSP6 (ΔSGF), identified in multiple SARS-CoV-2 variants of concern, was able to promote ER zippering even though NSP6 was linked to NSP7, indicating a selective mechanism that may compensate for slow proteolytic processing (30). Importantly, Bharadwaj *et al.* showed the highest predicted percentage aggregation propensity (PPAP) value for NSP6 among all NSPs (31). They also showed a rapid aggregate formation from aggregation prone region of NSP6 (residues 91–112) *in vitro* compared to NSP11, which was also predicted to form aggregates (31). On the other hand, NSP7 is a small, conserved nonstructural protein that functions primarily as a cofactor in viral RNA synthesis (32, 33). In the absence of NSP12 (RNA-dependent RNA polymerase), SARS-CoV NSP7 forms a hexadecameric complex with NSP8 that encircles double-stranded viral RNA (34). Within this assembly, NSP8 displays primer-dependent polymerase activity, while NSP7 enhances NSP8 binding to dsRNA and stabilizes the complex (34). However, due to the inherent low fidelity and processivity of NSP8, slow dissociation of NSP7 and NSP8 in SARS-CoV limits formation of the hexadecameric ring and favors primase activity over polymerase function (32). Together, these observations suggest that the slow cleavage of the NSP6–7 site by M^pro^ may reflect regulated processing rather than simple catalytic inefficiency. Therefore, understanding the structural basis for this slow processing could provide new insights into M^pro^ substrate specificity.

Here, we investigated the structural and biochemical basis for the slow processing of the SARS-CoV-2 NSP6–7 by M^pro^. We first compared the dynamics of dimeric M^pro^ bound to either NSP4–5 substrate peptide (hereafter referred to as NSP4–5) or NSP6–7 using three independent 250-ns all-atom, explicit-solvent MD simulations. This analysis revealed increased flexibility and altered positioning of the C-terminal residues of NSP6–7 within the M^pro^ active site. Guided by these observations, we generated NSP6–7 mutants in which the P2′, P3′, or P4′ residue was individually replaced with the corresponding residue from the faster-cleaved NSP4–5. DuProSense-based assays identified the P3′ M7F substitution as sufficient to enhance M^pro^-mediated NSP6–7 cleavage in living cells and reduce the apparent *K*_m_ *in vitro*. We further show that this substitution alters M^pro^ conformational dynamics by stabilizing the D187-R40 salt-bridge interaction and modulating residue-level communication within the protease. These findings provide a mechanistic basis for the slow processing of NSP6–7 and highlight the contribution of P3′ residue to M^pro^ activity and perhaps, substrate specificity.

## Results and discussion

### C-terminal residues of NSP6–7 are more flexible in the M^pro^ catalytic site than those in NSP4–5

To understand the mechanisms underlying the slower cleavage of NSP6–7 in comparison to NSP4–5, we first compared the amino acid sequence of the two peptides (Fig. 1*A*). While both peptides share conserved residues at the P4 (A residue), P1 (Q residue), and P1′ (S residue), they differ at the rest of the five positions out of the eight residues. These differences include V and T residues at the P3 position, L and V residues at the P2 position, G and K residues at the P2′ position, F and M residues at the P3′ position, and R and S residues at the P4′ position in NSP4–5 and NSP6–7, respectively (Fig. 1*A*). We posit that these differences in the amino acid sequence of NSP6–7, compared to NSP4–5, likely impacts the interaction of the peptide with the M^pro^ catalytic site and, in turn, affects its rate of cleavage. Therefore, to investigate the structural basis underlying the slower cleavage of NSP6–7, we compared the structural dynamics of M^pro^ in complex with either NSP6–7 or NSP4–5 using molecular dynamics (MD) simulations. For this, we generated structural models of dimeric M^pro^ in complex with either NSP4–5 or NSP6–7 using Modeller (35). We decided to utilize structural models generated using MODELLER to ensure placement of the substrate peptides within the M^pro^ active site as observed in an experimentally derived structure of the protein, and to provide reliable starting structures for MD simulations. The models were generated using the dimeric M^pro^ structure (PDB ID: 7TA4) as the template and were validated using DOPE scores (36), Ramachandran plot analysis using PROCHECK (37, 38), and visual inspection in PyMOL (39), confirming correct positioning of the substrate peptides in the M^pro^ dimer catalytic sites (Table S1; Figs. S1 and S2).

**Figure 1:**
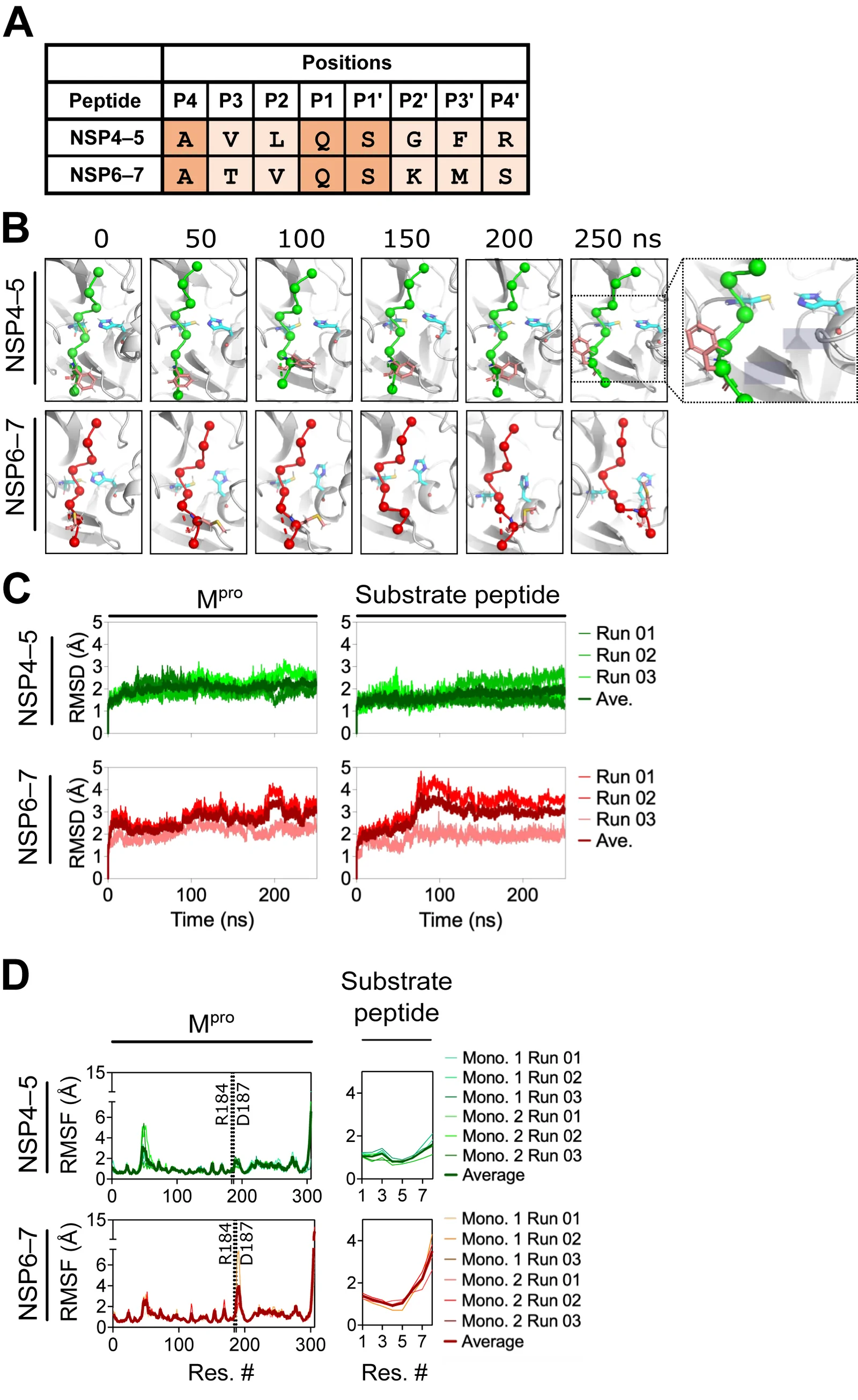
**MD simulations revealed a greater flexibility of the C-terminal residues ofNSP6**–**7bound to the M^pro^ catalytic site.***A*, amino acid sequences of NSP4–5 and NSP6–7 from the viral polyproteins pp1a and pp1ab. Sequence variation is highlighted through background coloring from *dark* (*lowest variation*) to *light* (*highest variation*) *orange*. *B*, snapshots of M^pro^ catalytic site with indicated substrate peptides (cartoon representation with Cα atoms shown as spheres) captured every 50 ns, obtained from a representative 250 ns long, all-atom, explicit solvent MD simulation showing the orientation of the substrate peptides over the course of the simulation. The cyan-colored residues correspond to the catalytic dyad (H41 and C145), while the brick-red side chain indicates the P3′ residue of the substrate peptide, as shown in the inset on the *right*. *C*, graphs showing RMSD of M^pro^ (*left panel*) and bound substrate peptide (*right panel*) in complex with NSP4–5 (*top panel*) and NSP6–7 (*bottom panel*) obtained from three independent, 250 ns all-atom explicit solvent MD simulation trajectories. *D*, graphs showing the RMSF values of M^pro^ with NSP4–5 complex (*top panels*; *right panel* for M^pro^ and *left* for NSP4–5) and M^pro^ with NSP6–7 complex (*bottom panels*; *right panel* for M^pro^ and *left* for NSP6–7), determined from a 250 ns long, all-atom, explicit solvent MD simulation. Residues R184 and D187 are highlighted with *dotted lines*.

**Figure 2:**
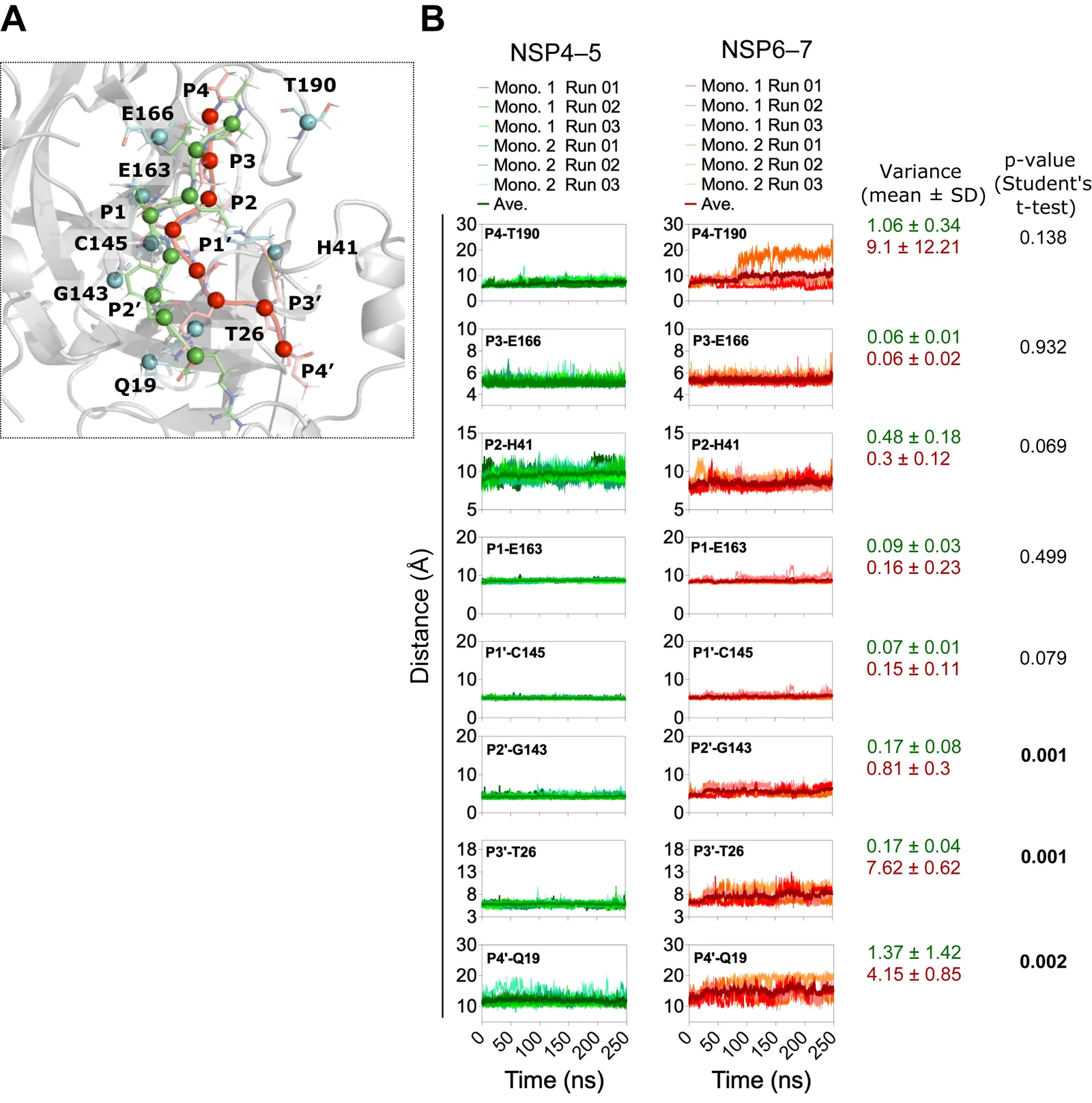
**MD simulations revealed an increased distance fluctuation ofNSP6**–**7C-terminal residues with M^pro^ catalytic site residues.***A*, cartoon representation of the M^pro^ catalytic subsite key residues (*cyan*) and NSP4–5 (*green*), and NSP6–7 (*red*). Cα atoms are depicted as spheres, and side chains are shown as sticks. *B*, graphs showing the distances measured between the indicated residues of the substrate peptides and the catalytic subsite residues M^pro^, obtained from 250 ns all-atom explicit solvent MD simulations. The *Left panels* display the distance over the simulation time of the indicated substrate peptides. Variance values (mean ± SD) in *green* and *red* are for NSP4–5 and NSP6–7, respectively, from three independent MD simulations of the dimeric M^pro^, and *p*-values shown are obtained from unpaired, two-tailed Student’s *t* test of the same values.

**Figure 3:**
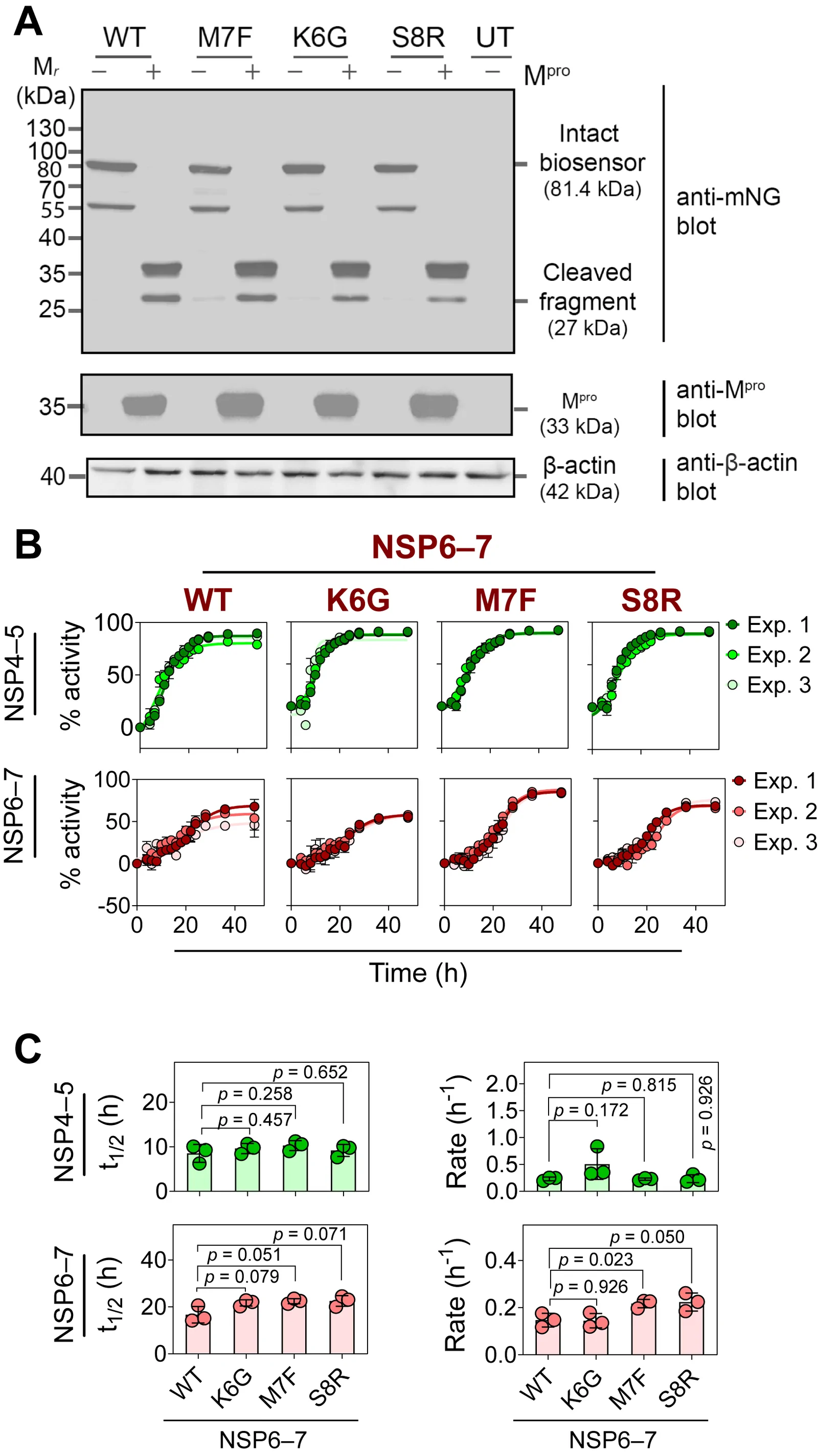
**Change in cleavage kinetics of NSP6**–**7 upon C-terminal residue mutations monitored in live-cell assay.***A*, Western blot analysis reveals the cleaved products of DuProSense biosensor containing NSP6–7 WT, NSP6–7(K6G), NSP6–7(M7F), and NSP6–7(S8R) upon M^pro^ expression. Lanes compare lysates from HEK293T cells expressing the DuProSense biosensor alone (no protease; first lane) and co-expressing M^pro^ (second lane), showing the conversion of full-length DuProSense biosensors (∼84 kDa) to a lower molecular weight band (CP bands) (∼27 kDa) upon M^pro^ expression. Predicted molecular weights for CP bands (kDa) are indicated on the *right* side. M^pro^ expression was confirmed by immunoblotting with anti-M^pro^ antibody, and loading was verified using anti-β-actin antibody. The additional bands detected at ∼35 kDa and ∼55 kDa in the anti-mNG blot are attributed to residual M^pro^ band signal after membrane stripping and a non-specific band detected in the absence of M^pro^, respectively. *B*, graphs showing the percentage protease activity of M^pro^ in cells expressing DuProSense biosensors containing WT and indicated mutant NSP6–7 at the indicated time points after transfection. *C*, graphs showing the half-time (t_1/2_) (*left panel*) and cleavage rate (*right panel*) of the M^pro^ activity in cleaving NSP4–5 measured from the living HEK293T cells expressing DuProSense biosensors containing WT and indicate mutant NSP6–7 with M^pro^. Data shown are mean ± SD from three independent experiments, with each experiment performed in triplicate. *p*-values shown were obtained from an unpaired, two-tailed Student’s *t* test.

**Figure 4:**
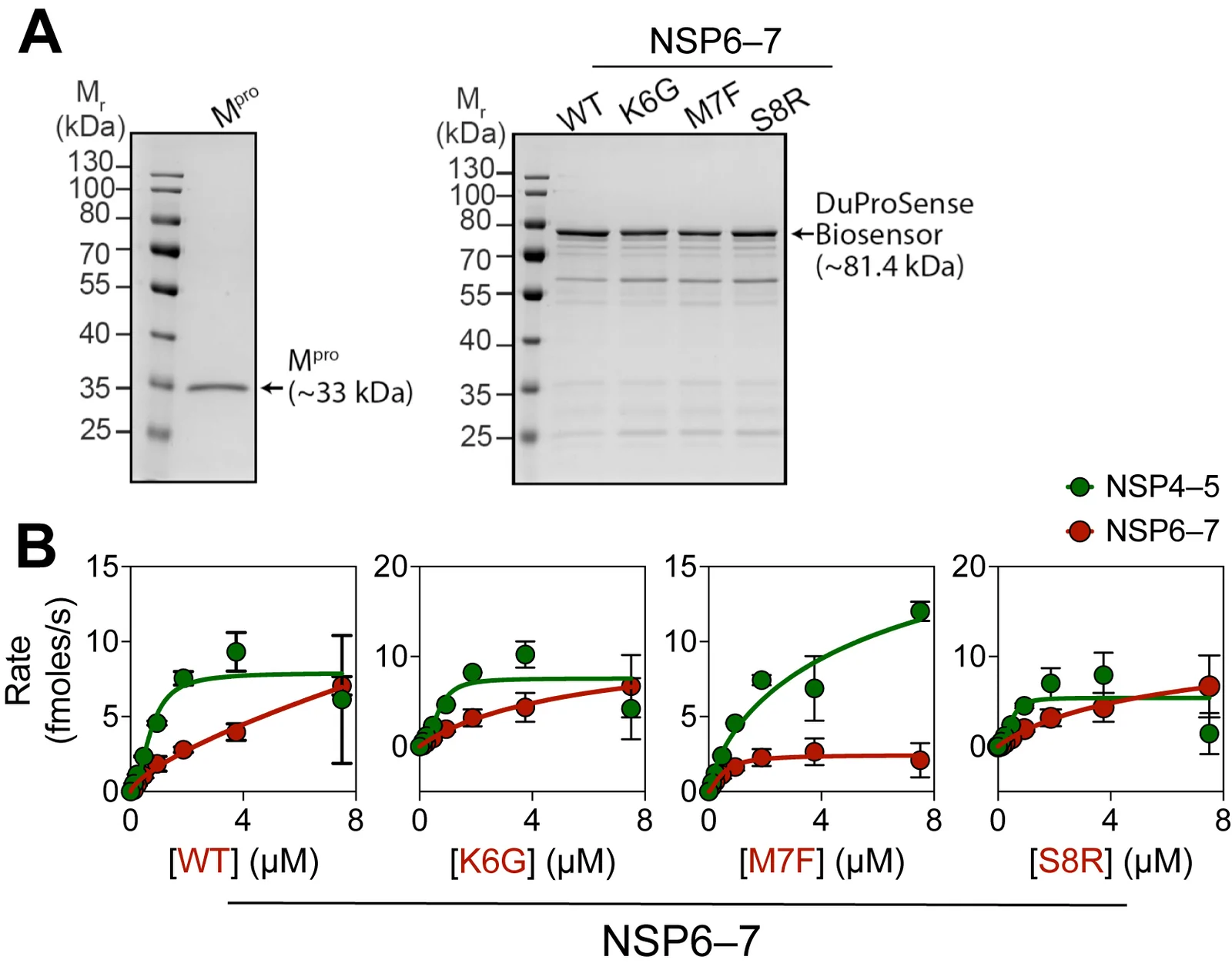
**Change in cleavage kinetics of NSP6**–**7 upon C-terminal residue mutations monitored in the *in vitro* assay.***A*, SDS-PAGE gel images showing the recombinantly purified SARS-CoV-2 M^pro^ (*left panel*) and DuProSense biosensors containing WT and mutant NSP6–7 (*right panel*). *B*, graphs showing the *in vitro* cleavage rates of NSP6–7 at varying substrate concentrations determined from enzyme kinetics assays using recombinantly purified DuProSense biosensors containing WT or mutant NSP6–7 and M^pro^.

We then performed three independent, 250 ns-long, all-atom MD simulations of M^pro^ in complex with either NSP4–5 or NSP6–7 using the NAMD 2.13 software (40) and analyzed the trajectories using VMD (41) and other tools. Visual inspection of the trajectories using PyMOL (39) revealed both NSP4–5 and NSP6–7 to be bound within their respective catalytic sites in M^pro^ throughout the MD simulation trajectories (Fig. 1*B*). However, while NSP4–5 maintained a stable binding pose, the C-terminal P3′ residue of NSP6–7 moved toward the H41 residue in the M^pro^ catalytic site (Fig. 1*B*). Comparison of the Cα-Cα distances between H41 and the C-terminal residues (P2′–P4′) of NSP4–5 and NSP6–7 revealed a significant reduction in distance for only the P3′ residue, suggesting a localized alteration in substrate positioning within the M^pro^ substrate-binding pocket (Figs. 1*B* and S3). We assessed the root-mean-square deviation (RMSD) of M^pro^ in both complexes over the course of the simulations. In all three independent trajectories, the RMSD of the M^pro^ dimer initially increased as the system relaxed from the starting conformation and subsequently reached a plateau, indicating the absence of large-scale conformational changes during the simulations (Fig. 1*C*, left panels, Fig. S4). A similar behavior was observed for both NSP4–5 and NSP6–7, which showed an initial increase in RMSD followed by plateauing (Fig. 1*C*, right panels).

Next, we performed the root mean square fluctuation (RMSF) analysis of Cα atoms to compare the residue-level flexibility of M^pro^ and the substrate peptide residues in the two complexes. The M^pro^ dimer in both complexes showed similar overall fluctuations; however, the RMSF values of residues R184–D187 were observed to be higher in M^pro^ bound with NSP6–7 (Fig. 1*D*, left panels; Fig. 5*A*, right panels; Table S5). We note that this region corresponds to the linker between domains II and III of M^pro,^ and increased fluctuations may influence interdomain communication in the M^pro^ dimer. Additionally, this region has been reported to have an important role in M^pro^ dynamics and nirmatrelvir binding interactions (42, 43). The differences in fluctuations of this linker region between the M^pro^-NSP4–5 and M^pro^-NSP6–7 complexes suggest that substrate-specific conformational changes in M^pro^ could influence the interaction of nirmatrelvir with M^pro^ as well as its potency. We have previously observed a higher IC_50_ value of nirmatrelvir in inhibiting M^pro^ in the presence of NSP6–7 as compared to NSP4–5 (16). On the other hand, RMSF analysis of NSP4–5 and NSP6–7 revealed that the residues at P1 and P1′ positions exhibited relatively lower RMSF values than terminal residues, indicating higher stability of these residues throughout the simulation trajectories (Fig. 1*D*, right panels). Importantly, the scissile bond, which is the specific peptide bond present in the substrate peptide that is cleaved by M^pro^, is located between the P1 and P1′ residues, indicating their structural rigidity in both M^pro^-substrate peptide complexes (44). Additionally, while the C-terminal residues were found to be more flexible than the N-terminal ones in both NSP4–5 and NSP6–7, the C-terminal residues in NSP6–7 showed higher flexibilities than those in NSP4–5 (Fig. 1*D*, right panels; Fig. S6).

To get a deeper insight into the dynamics of the C-terminal residues of NSP6–7 compared to those in NSP4–5 over time, we measured the distance of each of the substrate peptide residues with residues close to them from corresponding M^pro^ catalytic site, namely, P4-T190, P3-E166, P2-H41, P1-E163, P1′-C145, P2′-G143, P3′-T26, and P4′-Q19 (Fig. 2*A*). The distances were determined for each peptide and their corresponding M^pro^ monomer residues of M^pro^ dimer, and over the entire duration of the three independent MD simulations, and thus, resulted in a total of six distance traces for each residue pair. Further, we determined the variance of the distances measured over time from the six distance traces. This analysis revealed a generally constant distance for each of the NSP4–5 and M^pro^ catalytic site residues, except for P2-H41 and P4′-Q19 residue pairs (Fig. 2*B*). On the other hand, only P3-E166, P1-E163, and P1′-C145 residue pairs showed constant distance while the P4-T190, P2-H41, P2′-G143, P3′-T26, and P4′-Q19 residue pairs showed an increase in their distances in the case of NSP6–7 (Fig. 2*B*). Interestingly, this analysis also revealed a higher fluctuations in the distance between N and C-terminal residues of both NSP4–5 and NSP6–7 against the key M^pro^ catalytic site residues, while the residue pairs P1-E163 and P1′-C145 displayed minimal distance fluctuations in both M^pro^-NSP4–5 and M^pro^-NSP6–7 (Fig. 2*B*). We note that while NSP4–5 and NSP6–7 differ in their amino acid sequence at the N- and C-termini, the P1 and P1′ positions in both peptides are identical *i.e.* Q at P1 and S at P1′ position (Fig. 1*A*), which might explain the consistency in the distance measurements of these residues. Importantly, distance fluctuations analysis, determined as the variance of the distances measured over the MD simulation trajectories, of the C-terminal P2′-G143, P3′-T26 and P4′-Q19 residue pairs in NSP6–7 showed significantly higher variances in their distances, as compared to equivalent positions in NSP4–5 (Fig. 2*B*). Together, the RMSF and distance fluctuation analysis of the dimeric M^pro^ in complex with NSP4–5 and NSP6–7 suggest that the residues at the C-terminus of NSP6–7 shows a greater fluctuation and thus, affect its interaction with M^pro^ catalytic site residues, and which, in turn, likely impacts its M^pro^-mediated rate of cleavage.

### Increase in cleavage kinetics of M7F mutant NSP6–7 monitored in live cell and *in vitro* assays

Higher fluctuations in the distance between the C-terminal residues of NSP6–7 from the respective M^pro^ catalytic subsite residues in MD simulation analysis, as compared to NSP4–5, reported above could potentially contribute to its slower cleavage by M^pro^. We, therefore, aimed to evaluate if mutations in these C-terminal residues in NSP6–7 to those in NSP4–5 can increase its rate of cleavage by M^pro^. For this, single-point mutants of NSP6–7 were generated by replacing residues at position P2′, P3′, and P4′ with the corresponding residues from NSP4–5. Further, we utilized our recently reported two-color, Bioluminescence Resonance Energy Transfer (BRET)-based dual protease biosensor (DuProSense), containing NSP4–5 and NSP6–7, for monitoring cleavage of the two substrate peptides (16). Briefly, DuProSense was engineered to contain the mNeonGreen (mNG) (45) green fluorescent protein as the green channel BRET acceptor at the N-terminal, the NanoLuc (NLuc) (46) luciferase protein as the BRET donor in the middle and the mScarlet (mSca) (47) red fluorescent protein as the red channel BRET acceptor at the C-terminal. NSP4–5 and NSP6–7 were sandwiched between mNG and NLuc and NLuc and mSca, respectively. Bioluminescence spectral measurement and utilization of empirically derived expressions optimized for DuProSense allow unambiguous assignment of green and red channel BRET efficiency of the biosensor (16). M^pro^-mediated cleavage (16, 48, 49, 50, 51, 52, 53) of NSP4–5 and NSP6–7 in the DuProSense could be detected as decreases in the green and red channel BRET efficiency, respectively. We introduced single-point mutations in NSP6–7 and generated the K6G, M7F and S8R mutants (which we refer to as mutant NSP6–7 DuProSense biosensors in the following sections). Importantly, the presence of NSP4–5 in all the DuProSense biosensors allows internally controlled measurement of the M^pro^-mediated proteolytic cleavage of NSP6–7 in both living cells as well as *in vitro*.

We then first monitored the cleavage kinetics of the DuProSense biosensors containing WT and mutant NSP6–7 in live cells. For this, we transfected HEK293T cells with DuProSense biosensor containing WT and mutant NSP6–7 either without M^pro^ or along with M^pro^. To confirm M^pro^-mediated cleavage of these DuProSense biosensors containing WT and mutant NSP6–7, lysates collected from cells co-transfected with the DuProSense biosensors and M^pro^ were analyzed through Western blot analysis using antibodies against mNG, which is located at the N-terminus of the biosensor and report either intact or cleaved biosensor, and an antibody against M^pro^, which reports the expression of the protease (Figs. 3*A* and S5). Lysates prepared from cells transfected with only the DuProSense biosensor plasmids revealed a band at approximately 81.4 kDa, corresponding to the full-length, intact DuProSense biosensors, when probed with the anti-mNG antibody (Fig. 3*A*). However, lysates prepared from cells co-transfected with biosensor and an M^pro^ expressing plasmids revealed a band at ∼27 kDa when probed with the anti-mNG antibody, suggesting the cleavage of the biosensors and release of the mNG protein from the biosensor. Western blot also revealed a band at 33 kDa, corresponding to M^pro^, in the biosensor and M^pro^ co-transfected cells when probed with an anti-M^pro^ antibody (Fig. 3*A*), indicating an M^pro^-dependent cleavage of WT and mutant NSP6–7 containing DuProSense biosensors.

To monitor time-dependent cleavage of WT and mutant NSP6–7, we measured the bioluminescence spectra of cells expressing the biosensors in the absence or presence of M^pro^ over time. We then calculated BRET in the green (for NSP4–5) and red (for either WT or mutant NSP6–7) channels from the bioluminescence spectra. The green and red channel BRET values in the absence presence of M^pro^ at different times were then utilized to determine time-dependent percentage cleavage activity of M^pro^ against either NSP4–5 or NSP6–7 (WT or mutants) (Fig. 3*B*). This analysis revealed a high level of M^pro^ cleavage activity against NSP4–5, and a relatively lower level (61.74 ± 9.54%) of M^pro^ activity against NSP6–7 in the DuProSense biosensor containing NSP4–5 and WT NSP6–7 (Fig. 3*B*), as reported previously (16). Similarly, high levels of M^pro^ cleavage activity were observed against NSP4–5 in the biosensors containing mutant NSP6-7. Protease activities measured from the green BRET channel were consistently high (Fig. 3*B*), indicating efficient cleavage of NSP4–5 in the biosensors. WT NSP6–7 showed lower cleavage at 48 h (61.74 ± 9.54%); however, among the three mutants, M7F exhibited higher cleavage (85.14 ± 1%) (Fig. 3*B*; Table S3).

Furthermore, we performed the kinetic sigmoidal fitting of the percentage M^pro^ cleavage activities over time to obtain two key parameters, t_1/2_ and cleavage rate. To account for any possible expression-dependent effects, we compared the kinetics of NSP4–5 across the DuProSense biosensor containing WT or mutant NSP6–7 and found similar t_1/2_ and cleavage rates (Fig. 3*C*), indicating that any observed kinetic differences amongst WT and mutant NSP6–7 were likely not due to differences in DuProSense expression. The t_1/2_ of NSP6–7 mutants remained similar to that of the WT NSP6–7 in live cells (Fig. 3*C*, left panel). However, the rate of cleavage of the NSP6–7(M7F) was found to be significantly increased compared to the WT NSP6–7 (0.22 ± 0.03 *versus* 0.15 ± 0.02 h^-1^; *p* = 0.023) (Fig. 3*C*, right panel; Table S3). These results suggest that the presence of an M residue at P3′ results in a slower cleavage of NSP6–7.

Following the observation of an increase in the cleavage rate of the M7F mutant NSP6–7 in live cells, we investigated whether these mutations impact the cleavage kinetics *in vitro* as well and identify potential biochemical reasons for the increased cleavage of the M7F mutant NSP6–7. For this, we performed *in vitro* enzyme kinetics assays using recombinantly purified DuProSense biosensors containing WT or mutant NSP6–7 with recombinantly purified M^pro^ (Fig. 4*A*). Red channel BRET values were used to determine the rate of cleavage of WT and mutant NSP6-7, and data were fitted to the Michaelis-Menten model to determine their *V*_max_ and *K*_m_. Among the three mutants, NSP6–7(M7F) showed a significant reduction in apparent *V*_max_ (2.91 ± 1.22 *versus* 15.47 ± 3.93 fmol/s; *p* = 0.006) and apparent *K*_m_ value (0.77 ± 0.66 *versus* 9.24 ± 3.58 μM; *p* = 0.016) compared to WT NSP6-7 (Fig. 4*B*; Tables S4 and S5). Although the M7F substitution increased substrate cleavage in living cells and reduced the apparent *K*_m_ *in vitro*, the estimated catalytic efficiency (*k*_cat_/*K*_m_) appeared to be lower than that of the WT NSP6–7, suggesting that the enhanced cleavage may not be solely explained by *in vitro* assay results. In contrast, the NSP6–7(S8R) mutant exhibited a higher apparent catalytic efficiency than the WT NSP6–7 *in vitro*. However, despite showing a trend toward increased cleavage in live-cell assays, the increase was not statistically significant. These observed discrepancies between live-cell cleavage rates and *in vitro* catalytic efficiencies may be due to potential differences in substrate accessibility, conformational dynamics, and folding state or oligomerization behavior of recombinant proteins as compared to those in living cells, and other cellular determinants not fully recapitulated *in vitro* (54, 55). For instance, continuous expression of the DuProSense biosensor and M^pro^, following transfection of cells with their respective plasmids, creates dynamic changes in the levels of the biosensor and M^pro^ protease in living cells. On the other hand, *in vitro* assays are performed with defined and high concentrations of recombinant proteins under biochemical conditions optimized for proteolytic activity of M^pro^. For instance, kosmotropic salts such as sodium citrate, included in the *in vitro* assays, have been reported to enhance M^pro^ activity, potentially through stabilization of the M^pro^ dimer (52, 56), while interactions with host-cell proteins and putative host substrates may influence M^pro^ activity in living cells (57, 58).

The P3′ position of M^pro^ substrate peptides showed a greater flexibility and less specificity than the conserved P1–P1′ region in the MD simulations. In some M^pro^ substrate peptides, for example, NSP9–10, the P3′ residue (often an N residue) is reported to form stabilizing contacts with residues T24 and T25 present in a proximity to the residue T26 in the M^pro^ catalytic site (23). Moreover, the C-terminal residues of M^pro^ substrate peptides, including the P3′ position, are more exposed to solvent and more variable in sequence than the conserved P4–P1′ region. Because of this, the P3′ position was previously considered highly tolerant and thought to contribute little to substrate binding or cleavage efficiency (15). However, results reported above demonstrate that the P3′ residue can significantly influence cleavage kinetics of NSP6–7, highlighting an important role of the P3' residue in M^pro^ substrate processing.

### M7F substitution reshapes M^pro^ structural dynamics and intramolecular communication pathways

Given that the M7F substitution at the P3′ position increased NSP6–7 cleavage in living cells and decreased apparent *K*_m_ *in vitro*, we next performed all-atom MD simulations of M^pro^ in complex with the NSP6–7(M7F) to determine whether the altered cleavage is associated with a dynamic profile resembling that observed for the faster cleaving NSP4–5 or with a distinct pattern of protease and substrate peptide dynamics. Accordingly, RMSF, substrate peptide–protease interface distance, dynamic cross-correlation (DCC) and residue network analyses were performed to compare the M^pro^ bound with NSP4–5, NSP6–7, and NSP6–7(M7F) through pairwise comparisons. RMSF analysis revealed a significant increase in theflexibility of 13 M^pro^ residues in the case of NSP6–7 complex, as compared to the NSP4–5 complex, including the three key substrate peptide binding-site residues N142, H164, and D187 in M^pro^, whereas the NSP6–7(M7F) complex showed increased flexibility in a total of 15 residues, but only one substrate peptide binding-site residue, namely N142, in M^pro^ (Figs. 5*A* and S6). Further, we observed that the M7F substitution at the P3′ position in NSP6–7 resulted in a change in the flexibility of a total of 60 M^pro^ residues that are distributed across all the three domains of M^pro^, including substrate peptide binding-site residues T45, M49, and D187. Importantly, the N142 residue consistently showed altered flexibility in both NSP6–7 and NSP6–7(M7F) complexes relative to the NSP4–5 complex (Figs. 5*A* and S6). In contrast, the D187 residue showed differential behavior, with increased flexibility in the NSP6–7 complex and reduced flexibility in the NSP6–7(M7F) complex, compared to the NSP4–5 complex. These observations suggest a potential role of the N142 residue in NSP4–5-specific M^pro^ structural dynamics, while the D187 residue could be associated with NSP6–7 driven M^pro^ structural dynamics (Figs. 5*A* and S6). We note that while M^pro^ residues showed substrate peptide specific changes in RMSF values, the substrate peptides themselves showed similar residue level fluctuations (Tables S7 and S8).

**Figure 5:**
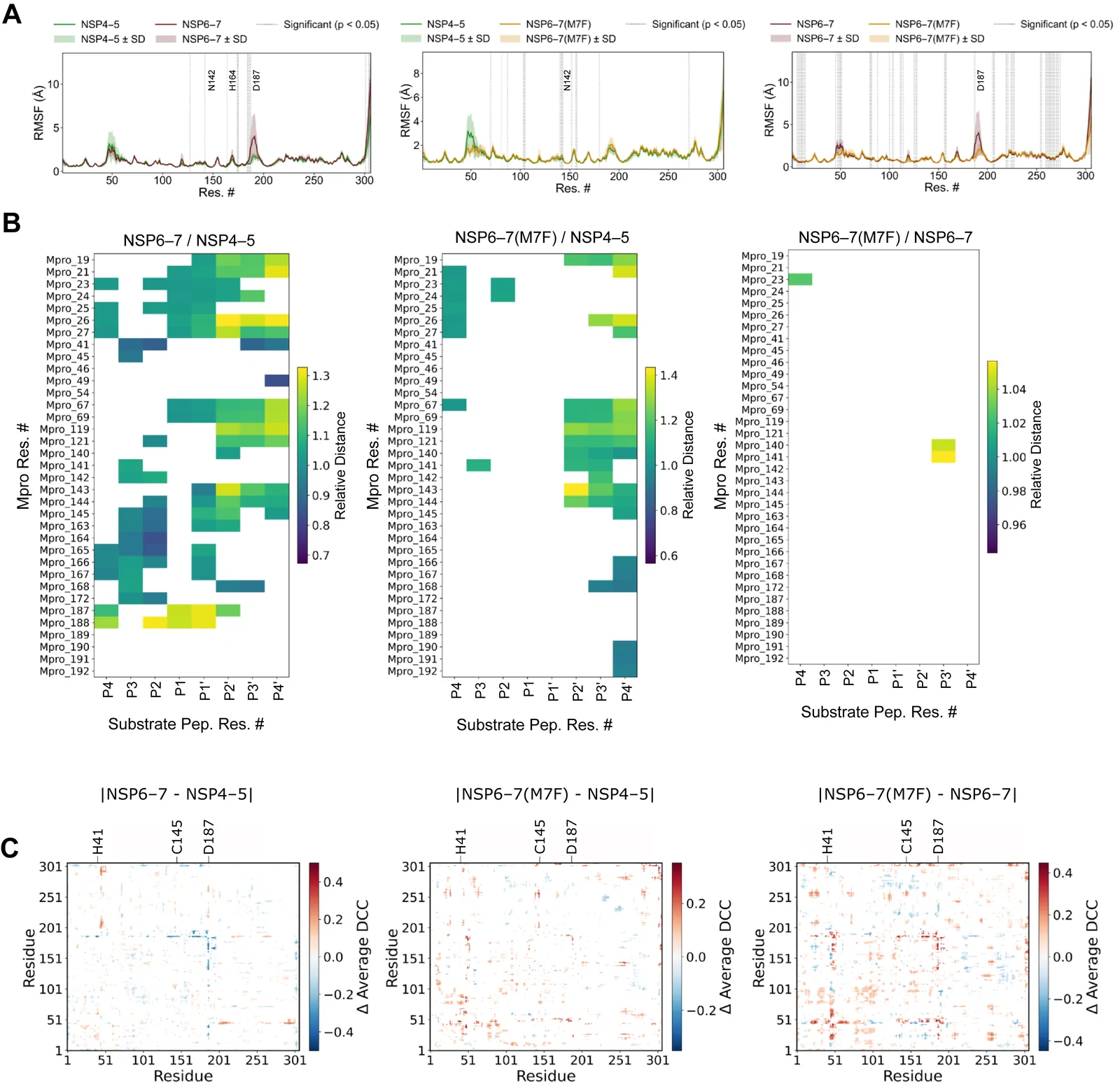
**Comparative structural dynamics and interface analysis of M^pro^ in complex with NSP4**–**5, NSP6**–**7, and NSP6**–**7(M7F).***A*, graphs showing RMSF of M^pro^ residues compared across complexes: NSP4–5 *versus* NSP6–7 (*left*), NSP4–5 *versus* NSP6–7(M7F) (*middle*), and NSP6–7 *versus* NSP6–7(M7F) (*right*). Shaded areas represent the standard deviation (SD). *Vertical dotted lines* highlight regions with statistically significant differences (*p* < 0.05). Key catalytic and binding pocket residues (*e.g.*, N142, H164, D187) are labeled. *B*, heatmaps showing relative average distances between substrate peptide residues (P4-P4′; *x-axis*) and selected M^pro^ residues (*y-axis*). The *left*, *middle*, and *right panels* show comparisons of NSP6–7 relative to NSP4–5, NSP6–7(M7F) relative to NSP4–5, and NSP6–7(M7F) relative to NSP6–7, respectively. The color scale represents the ratio of average distances between the indicated complexes. Only residue pairs showing statistically significant difference (*p* ≥ 0.05 obtained from an unpaired, two-tailed Student’s *t* test) are shown. *C*, difference dynamic cross-correlation (ΔDCC) matrices showing pairwise changes in correlated motions among M^pro^ residues. Each matrix was generated by subtracting the DCC values of the reference complex from those of the compared complex: NSP6–7 – NSP4–5 (*left*), NSP6–7(M7F) − NSP4–5 (*middle*), and NSP6–7(M7F) − NSP6–7 (*right*). Positive values represent increased residue-pair correlation in NSP6–7 or NSP6–7(M7F), depending on the comparison, whereas negative values represent decreased correlation or increased anti-correlated motion relative to the corresponding reference complex. White regions indicate residue pairs that did not show statistically significant differences (*p* ≥ 0.05). Positions of key residues (H41, C145, and D187) are indicated by line markers along the axis. Data shown are obtained from each monomer and three independent, 250 ns-long MD simulations, determined at 10 ns intervals. *p*-value was obtained from an unpaired, two-tailed Student’s *t* test analysis.

To assess whether the M7F substitution alters substrate positioning within the M^pro^ catalytic site, we compared average Cα-Cα distances between substrate peptide residues and M^pro^ catalytic site residues (Fig. 5*B*). For this, we measured the distances (Cα atom) between the residues over the trajectories for each monomer–substrate peptide pair, yielding six independent datasets across the three replicate 250 ns long simulations. All six monomer–substrate peptide datasets were used for statistical analysis to evaluate the significance of observed differences between complexes. First, comparison of NSP4–5 and NSP6–7 complexes revealed significant differences in the distance between several pairs of M^pro^-substrate peptide interface residues that are located close to the N- and C-terminal of the substrate peptides. In particular, the NSP6–7 complex showed reduced distances between multiple substrate peptide residues and the catalytic dyad residues H41 and C145 in M^pro^, including both the N-terminal (P2–P3) and the C-terminal (P3′–P4′) positions (Fig. 5*B*). On the other hand, comparison of the NSP4–5 and NSP6–7(M7F) complexes showed fewer and more localized differences in the interface residue distances. Specifically, changes in the interface residue distances were largely confined to the C-terminal P2′-P4′ position of the substrate peptide, involving L67, Q69, Y119, S121, and L140 residues in M^pro^, while no significant changes were observed for the catalytic dyad residues H41 or C145 in M^pro^. Finally, a direct comparison of the NSP6–7 and NSP6–7(M7F) complexes revealed largely similar interface distance profiles, with only minor local variations, including increased distances between the P3′ and residues L140 and N141 in M^pro^. Overall, the NSP6–7(M7F) complex maintained an interface residue distance profile more similar to the NSP6–7 complex than to the NSP4–5 complex (Fig. 5*B*).

Given the observed differences in residue flexibility and M^pro^-substrate peptide interface distances, we next examined how the M7F substitution influences correlated motions within M^pro^ in the NSP6–7(M7F) substrate peptide complex by analyzing DCC of residue movements in comparison to the NSP4–5 and NSP6–7 complexes (Figs. 5*C* and S7), which revealed differences in DCC between the three complexes. For comparison of DCC of the NSP4–5 complex with those of the NSP6–7 and NSP6–7(M7F) complexes, we plotted the differences between NSP6–7 and NSP6–7(M7F) complexes and the NSP4–5 complex while for comparison of DCC between NSP6–7 and NSP6–7(M7F) complexes, we plotted the difference in DCC of the NSP6–7(M7F) and NSP6–7 complexes (Fig. 5*C*). In comparison with NSP4–5, the NSP6–7 complex showed changes in correlated motions across several regions in the protein, with the most pronounced effect observed with the D187 residue, which exhibited a reduction in positive correlation with residues in domains I and II of M^pro^, including catalytic dyad residues H41 and C145 (Fig. 5*C*, left panel). However, these DCC differences were not observed in the NSP6–7(M7F) complex (Fig. 5*C*, middle panel). In addition, the NSP6–7(M7F) complex showed enhanced correlated motions within domain I relative to the NSP4–5 complex (Fig. 5*C*, middle panel). Interestingly, the NSP6–7(M7F) complex showed increased positive correlation of M^pro^ D187 residue with domains I and II residues, including the catalytic dyad residues H41 and C145, as compared to the NSP6–7 complex (Fig. 5*C*, right panel). Additionally, the C44–L50 residues exhibited increased positive correlation with domains I and II residues in the NSP6–7(M7F) complex relative to the NSP6–7 complex. We note that a significant reduction in RMSF values was also observed for residues T45 and E47–L50 in this region in the NSP6–7(M7F) complex, suggesting that the P3′ M7F substitution may contribute to reduced local flexibility and enhanced dynamic coupling of these residues with the core domains of M^pro^ (catalytic pocket is present at the interface of domain I and II). Together, these observations suggest that the M7F substitution may alter residue-level communication within M^pro^ residues.

To further investigate these differences between the various M^pro^-NSP substrate peptide complexes, average shortest path (L) analysis was performed to assess changes in residue accessibility and communication within M^pro^ in the NSP4–5, NSP6–7, and NSP6–7(M7F) complexes (Fig. 6*A*). The L value represents the mean number of shortest network paths connecting a residue to all other residues and provides an estimate of residue accessibility and efficiency of intra-protein communication, where changes in L (ΔL) reflect altered communication pathways within the protein. Although ΔL changes were observed for multiple residues, we focused our attention on those showing significant and consistent changes in both RMSF and DCC values. Notably, the D187 residue exhibited an increased ΔL value in the NSP6–7 complex relative to the NSP4–5 complex (NSP4–5 = 0.036 ± 0.008, NSP6–7 = 0.134 ± 0.06; *p* < 0.008), which may steer conformational changes in the protein (Fig. 6*A*, upper panel). This change was not observed in the NSP6–7(M7F) complex, where the D187 residue displayed a ΔL profile more similar to that of the NSP4–5 complex (Fig. 6*A*, middle panel, bottom panel). We note that the M^pro^ D187 residue has been reported previously to contribute to the organization of the conserved water network that stabilizes the zwitterionic Cys145^-^/His41^+^ catalytic state, thereby supporting catalytic dyad activation and substrate cleavage (59, 60, 61). In addition, the D187 residue forms a stabilizing salt bridge with R40, which helps preserve the active-site geometry and sustain the water-mediated network associated with the Cys145^-^/His41^+^ catalytic dyad (61). The functional importance of these residues is further supported by deep mutational scanning studies showing that R40 and D187 are highly mutation-intolerant, with substitutions at these positions frequently resulting in loss of M^pro^ function (62). In our MD simulations, C145 and H41 were modeled in their neutral, pre-catalytic states during CHARMM-GUI system preparation rather than the transient C145^-^/H41^+^ ion-pair state. We chose this with the aim to understand M^pro^–substrate peptide binding and conformational dynamics prior to the catalytic reaction. The same protonation states were applied across all complexes to ensure consistent comparison of structural dynamics.

**Figure 6:**
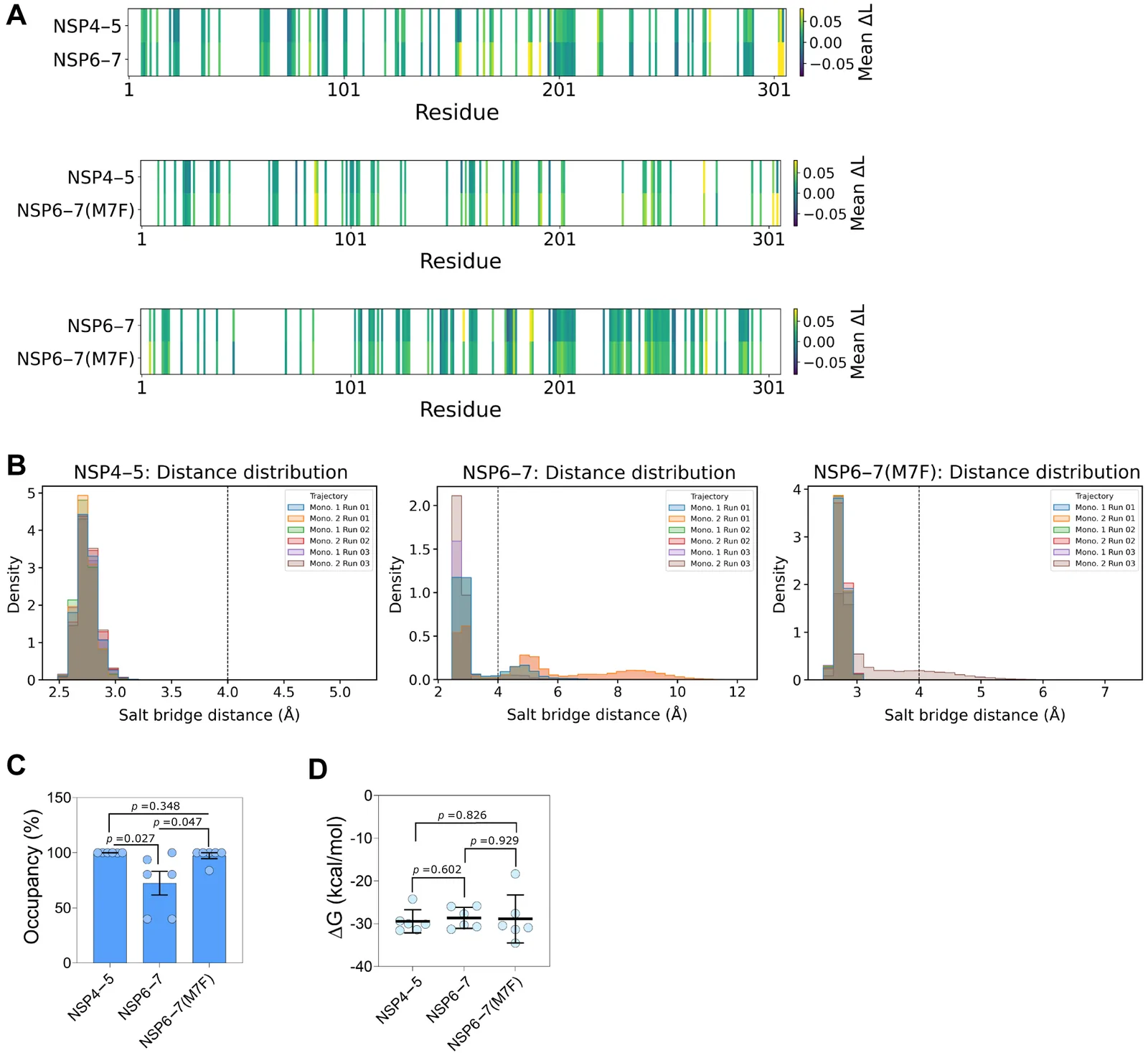
**Network communication analysis, salt-bridge dynamics, and binding free energy comparison across M^pro^–substrate peptide complexes.***A*, residue-specific mean change in average shortest path (ΔL) across M^pro^ residues (1–306) comparing NSP4–5 *versus* NSP6–7 (*top*), NSP4–5 *versus* NSP6–7(M7F) (*middle*), and NSP6–7 *versus* NSP6–7(M7F) (*bottom*). *B*, distance distribution profiles showing probability density of interatomic D187–R40 distances (Å) across MD trajectories for both protomers (Monomer 1 and Monomer 2) in NSP4–5 (*left*), NSP6–7 (*middle*), and NSP6–7(M7F) (*right*). The vertical dashed line indicates the 4.0 Å cutoff used to define salt-bridge formation during the analysis. *C*, bar plots showing percentage occupancy (%) of the monitored salt bridge in NSP4–5, NSP6–7, and NSP6–7(M7F) M^pro^-substrate peptide complexes. *D*, Binding free energy (ΔG) estimates (kcal/mol) for NSP4–5, NSP6–7, and NSP6–7(M7F) complexes. Data shown are obtained from each monomer and three independent, 250 ns-long MD simulations, determined at 10 ns intervals. *p*-value were obtained from unpaired, two-tailed Student’s *t* test analysis.

Given the critical role of the D187 residue in stabilizing the H41 residue through the R40 residue-anchored salt-bridge network and the observed substrate peptide-specific changes in the structural dynamics of the D187 residue reported above, we next examined whether binding of NSP6–7 to M^pro^ potentially affects the D187-R40 salt-bridge interaction (Fig. 6*B*). Distance distribution analysis of the D187-R40 residues revealed a highly stable salt-bridge in the NSP4–5 complex, with a narrow distribution centered in the range of 2.6 to 2.9 Å and minimal sampling beyond 3.2 Å (Fig. 6*B*, left panel). In contrast, the NSP6–7 complex showed a broad and bimodal distribution, with a minor population with distances in the range of 2 to 3.5 Å and a large population (non-contacting) with distances in the range of 4 to 12 Å, indicating frequent disruption of the salt bridge (Fig. 6*B*, middle panel). The NSP6–7(M7F) complex showed a largely concentrated distribution of distances ranging between 2.6 to 3.2 Å with a minor population of distances up to ∼7 Å, suggesting a comparatively more persistent D187-R40 interaction profile. Overall, the NSP4–5 complex maintained a highly stable salt bridge interaction with occupancy values consistently ∼99.9 to 100.0% across all three MD simulation runs. In contrast, the NSP6–7 complex showed a substantial reduction and variability in occupancy (ranging from 40.0 to 100% with a mean value of ∼72.4%), consistent with frequent disruption of the interaction as reflected in the broadened distance distribution (Fig. 6*C*). On the other hand, the NSP6–7(M7F) complex showed a highly stable interaction with near-complete occupancy (ranging from 83.8 to 100% with a mean value of ∼97.3%), approaching the stability observed in the NSP4–5 complex (Fig. 6*C*). The statistical analysis of D187-R40 salt-bridge occupancy showed a significant reduction in the NSP6-7 complex compared to the NSP4–5 complex (72.39 *versus* 99.96%; *p* = 0.0277), whereas the NSP6–7(M7F) complex showed an occupancy similar to that of the NSP4–5 complex (97.30 *versus* 99.96%; *p* = 0.348). Comparison of the NSP6–7 and NSP6–7(M7F) complexes revealed a higher D187-R40 salt-bridge occupancy in the NSP6–7(M7F) complex (97.30 *versus* 72.39%; *p* = 0.047) (Fig. 6*C*), suggesting a potential role for the D187-R40 salt-bridge in the increased cleavage of NSP6–7(M7F).

Given that the M7F substitution in NSP6–7 resulted in a decrease in the apparent *K*_m_ observed in the *in vitro* assays, we determined the binding free energy change (ΔG) of the complexes using the MM/PBSA method (50, 51, 63, 64). However, we observed similar ΔG values for binding of NSP4–5, NSP6–7, and NSP6–7(M7F) to M^pro^ (−29.44 ± 2.70, −28.64 ± 2.45, −28.87 ± 5.61 kcal/mol for NSP4–5, NSP6–7 and NSP6–7(M7F), respectively) (Fig. 6*D*). This suggests that the decreased apparent *K*_m_ of NSP6–7(M7F) may arise from factors not fully captured by the computational MD simulations and MM/PBSA analysis, such as altered association or dissociation kinetics, conformational selection, productive substrate positioning, or transition-state stabilization. Overall, the effects of the P3′ M7F substitution in NSP6–7 on M^pro^ structural dynamics were not limited to features similar to those observed in the NSP4–5 complex. While the alterations observed at the D187 residue in the NSP6–7(M7F) complex became more similar to those observed in the NSP4–5 complex, the mutant also showed additional, distinct changes that were not observed in either the NSP4–5 or the NSP6–7 complexes, including enhanced correlated motions and reduced flexibility within C44-L50 residues. These findings suggest that the increased cleavage rate in living cells and decrease in the apparent *K*_m_ observed with the M7F substitution may arise from a combination of NSP4–5-like dynamic features and mutation-specific changes in M^pro^ conformational dynamics.

## Conclusion

To conclude, this study focused on the structural dynamics of NSP6–7 in complex with the SARS-CoV-2 M^pro^ dimer to identify potential molecular interactions affecting the cleavage kinetics of this site. We show that sequence variation at the P3′ position critically modulates cleavage behavior, with the M7F substitution increasing live cell cleavage rate and altering *K*_m_ *in vitro*. These effects appear to be associated with alterations in D187-mediated dynamics and broader changes in M^pro^ conformational behavior, including NSP6–7(M7F)-specific rearrangements that resemble those observed in the NSP4–5 complex, along with additional distinct features. Overall, this study demonstrates that the P3′ residue can significantly influence M^pro^ substrate peptide cleavage kinetics, highlighting its important role in the interaction of M^pro^ with its substrate peptides.

## Experimental procedures

### Structural modeling and molecular dynamics (MD) simulations

The 3D structures of M^pro^ dimer with NSP4–5 and NSP6–7 were generated previously (64) using a homology-modeling tool, MODELLER (35). The structure of the C145A mutant M^pro^ dimer in complex with the NSP9–10 substrate peptide (PDB ID: 7TA4) (8) was obtained from the RCSB database (https://www.rcsb.org) and used as the template. Because the template structure (PDB ID: 7TA4) contains the C145A mutation in the catalytic site, the wild-type catalytic residue was restored by substituting A145 with C145 during model generation. Additionally, the NSP9–10 was replaced with NSP4–5 (AVLQSGFR) or NSP6–7 (ATVQSKMS) or NSP6–7(M7F) (ATVQSKFS) to generate the respective complex. Quality of the generated models was assessed using the Discrete Optimized Protein Energy (DOPE) score, a statistical potential that estimates the relative stability of protein structures, where lower scores indicate more thermodynamically favorable conformations (Table S1) (36). Further, structural geometry of the models was evaluated using Psi/Phi Ramachandran plots generated with PROCHECK (https://saves.mbi.ucla.edu) (37, 38) (Fig. S1). Secondary structural elements and substrate peptide placement were visually inspected using PyMOL (39) to verify proper folding and structural integrity. Together, these analyses demonstrated that the models were of high quality and suitable for subsequent MD simulations. Additionally, M^pro^-substrate peptide complex structures were also generated using the AI-based protein structure prediction tool AlphaFold2 (65). However, the substrate peptide regions exhibited lower prediction confidence, as reflected by reduced pLDDT scores and increased conformational variability. To compare the two modeling approaches, the RMSD between the substrate peptide conformations generated by MODELLER (35) and AlphaFold2 (65) was calculated using PyMOL (39), and the substrate peptide showed noticeable differences compared to MODELLER (Fig. S2) and was associated with low confidence scores in these regions (mean pLDDT = 37.72 ± 15.58), in contrast to the high-confidence M^pro^ dimer core (mean pLDDT = 89.11 ± 3.6; Table S2).

The topology and parameter files for MD simulations were prepared using the CHARMM-GUI Solution Builder input generator (66) employing the CHARMM36m force field (67) for all M^pro^-substrate peptide complexes. Briefly, the solution builder was selected to generate a protein solution system in an aqueous solvent system with a water box size fit to protein size and edge distance of 10 Å. Na^+^ and Cl^-^ ions at a concentration of 0.15 M were added using the Monte Carlo ion placing method. The equilibration input files were generated by an NVT ensemble molecular system at 310 K. The thermal equilibration was performed using NAMD 2.13 (68) and was followed by three independent, 250 ns long production runs. Initial 100 ns MD simulation runs for the NSP4–5 and NSP6–7 M^pro^ complexes have been reported previously (64). Simulation trajectories were analyzed for Cα-based RMSD, Cα-based RMSF, and inter-residue side-chain atom distances using VMD (41) as described in previous reports (53, 69, 70). To further assess conformational sampling, two-dimensional free-energy landscapes were generated using RMSD and radius of gyration (RoG) as collective variables. Each replicate simulation was initiated using independent initial velocity seeds, enabling statistically independent trajectories for analysis. Distinct features observed in the resulting free-energy landscapes across replicates further reflect independent sampling of conformational space (Fig. S2). The average distance was determined by calculating the average of the measured inter-residue distances over the simulation time. To quantify the salt bridge interaction between residues D187 and R40, we performed distance distribution analysis between the D187 and R40 side chains, specifically measuring the minimum heavy-atom distances between ASP(D) carboxylate oxygen atoms (OD1/OD2) and Arg (R) guanidinium nitrogen atoms (NH1/NH2/NE) across molecular dynamics trajectories. A salt bridge was defined using a 4.0 Å cutoff, and occupancy was calculated as the percentage of trajectory frames. Residue interaction network analysis (L) and dynamic cross-correlation (DCC) analysis, based on the positional fluctuations of Cα atoms, was performed using the MD-TASK software suite (71). The binding free energy (ΔG) was estimated using the Molecular Mechanics Poisson–Boltzmann Surface Area (MM-PBSA) approach (50, 51, 63, 64) implemented through the CaFE 1.0 plugin (72) in VMD. ΔG values were calculated for successive 10-ns windows across the 250-ns trajectories using frames sampled at 2-ns intervals.

### Plasmid generation, cell culture, and transfection

DuProSense biosensors containing NSP6–7 mutants were generated by replacing NSP6–7 sequence of previously generated DuProSense containing M^pro^ NSP4–5 and NSP6–7 plasmid (16) (Table S2) (GenScript Biotech (Singapore) Pte. Ltd, Singapore). HEK293T cells were used to perform both live cell assays and for preparing DuProSense biosensor containing cell lysates for Western blot. Briefly, cells were maintained in DMEM supplemented with 10% FBS and 1% penicillin–streptomycin at 37 °C with 5% CO_2_. For live cell BRET assays, HEK293T in 96-well white plates were transfected using polyethyleneimine (PEI) lipid (Sigma-Aldrich; 408727-100mL) after 24 h of seeding. For determining the temporal live cell cleavage kinetics, HEK293T cells were co-transfected with DuProSense biosensor containing WT mutant NSP6–7 and pLVX-EF1alpha-SARS-CoV-2-nsp5-2×Strep-IRES-Puro (M^pro^ WT) (a gift from Nevan Krogan; Addgene #141370; http://n2t.net/addgene:141370; RRID:Addgene 14137) at a ratio of 1:5 of biosensor-to-protease plasmid DNA (73). Bioluminescence spectral scans were acquired according to the previously described protocol (48, 49, 50, 51, 52, 53).

### Live cell protease activity assay

HEK293T cells were transfected with relevant plasmid DNA in 96-well white flat-bottom plates (16, 51, 52, 74, 75). For spectral characterization and protease activity monitoring, BRET measurements were performed 48 h post-transfection by addition of furimazine (Promega; catalog number: N1571) diluted 1:200 according to the manufacturer's recommended preparation protocol. For live-cell temporal cleavage kinetic assays, bioluminescent spectral measurements were performed at the indicated points post-transfection (2, 4, 6, 8, 10, 12, 24, 36, and 48 h) following addition of furimazine at a 1:200 dilution (16). Three independent experiments were performed in triplicate. The BRET values were calculated using empirically derived dual-color BRET equations described in an earlier study by Fatima *et al.* (16). Briefly, the green and red channel BRET signals were calculated from the luminescence intensities using the following equations:

$${BRET}_{green}=\frac{{\;I}_{533}+{\;I}_{615}\;}{\;{\;I}_{467}\;}\;{and\;BRET}_{red}=\frac{{\;I}_{615}}{{\;I}_{467}+{\;I}_{533}+{\;I}_{560}}$$

The percentage protease activity was calculated relative to the no-protease control using the following equation:

$$\%\;protease\;activity=\frac{{BRET}_{No\;Protease}-{BRET}_{Protease}}{{BRET}_{No\;Protease}}\times 100$$

The temporal cleavage kinetics of the live-cell protease assay were analyzed by fitting the percentage protease activity values to a sigmoidal kinetic model in GraphPad Prism (version 9, GraphPad Software; www.graphpad.com). The equation used for fitting was:

$$\%\;protease\;activity=baseline\;\%\;protease\;activity+\frac{maximum\;\%\;protease\;activity}{1+\exp\left(-k\left(time-t_{\frac{1}{2}}\right)\right)}$$

where *k* represents the cleavage rate constant and *t*_*1*_*_/_*_*2*_ represents the time required to reach half-maximal protease activity.

### Western blot analysis

HEK293T cells co-transfected with the relevant plasmid DNA were lysed in 200 μl of 2× Laemmli sample buffer (heated to 95 °C and sonicated before addition). Western blot was carried out as previously described (51, 52). Blots were incubated with anti-mNeonGreen antibody (anti-mNeonGreen rabbit polyclonal antibody; Proteintech-29523-1-AP; dilution, 1:5000) overnight at 4 °C in dilution buffer (TBS-T containing 5% BSA) and SARS-CoV-2 3C-Like Protease Antibody (Polyclonal antibodies; Cell Signaling TECHNOGY-51661; dilution, 1:5000). Secondary anti-mouse IgG HRP (Anti-Mouse Ig: HRP Donkey pAb; ECM biosciences- MS3001; dilution, 1:20,000) and anti-rabbit IgG HRP (Anti-Mouse Ig: HRP Mouse mAB; ECM biosciences-RS3251; dilution, 1:20,000) were used for probing cleaved DuProSense biosensor proteins and M^pro^, respectively.

### Protein expression and purification

The BL21-CodonPlus (DE3) *E. coli* strain was transformed with the SARS-CoV-2 M^pro^ expression plasmid pETM33_NSP5_M^pro^ (a gift from Ylva Ivarsson; Addgene plasmid #156475; http://n2t.net/addgene:156475; RRID:Addgene_156475 (76),was cultured in Luria Broth (LB) medium supplemented with 50 μg/ml kanamycin and chloramphenicol and incubated overnight at 37 °C with shaking at 220 rpm. The overnight culture was inoculated into fresh LB medium and grown for 2 h at 37 °C, 220 rpm, followed by induction with 1 mM IPTG for 2.5 h under the same conditions. Cells were pelleted (4000×*g*, 10 min, 4 °C), resuspended in 10 ml lysis buffer (50 mM Tris pH 8, 300 mM NaCl, 10 mM β-ME, 1 mM PMSF, 10% glycerol), and sonicated on ice for 30 min. The lysate was clarified by centrifugation (18,000×*g*, 1 h, 4 °C) and the supernatant was incubated with GSH beads for 2 h. Beads were washed with buffer (50 mM Tris pH 7, 150 mM NaCl, 10 mM β-ME, 1 mM EDTA, 10% glycerol, 0.01% Triton X-100) and bound M^pro^ was cleaved overnight at 4 °C using PreScission Protease (GE Healthcare, #27-0843-01) in cleavage buffer. The released SARS-CoV-2 M^pro^ was collected by centrifugation (500×*g*, 4 °C), aliquoted, and stored at −80 °C (52, 74). DuProSense biosensors containing WT or mutant NSP6–7 were expressed in the bacterial expression system, purified using Ni-NTA beads (HisPur Ni-NTA Resin, Thermo Fisher Scientific, #88221) and concentrated using Pierce Protein Concentrators PES (Thermo Fisher Scientific, #88527, 10K MWCO).

### *In vitro* enzyme kinetics assay

*In vitro* enzyme kinetics assays were performed in an assay buffer (TBS containing 1 M sodium citrate, 2 mM DTT & 1 mM EDTA) optimized for M^pro^ protease activity at 37 °C using recombinantly purified DuProSense biosensors at varying concentrations, with reactions initiated by the addition of recombinantly purified M^pro^ (200 nM) to the DuProSense biosensor buffer mixture, which was incubated for 60 min for M^pro^ at 37 °C. The reaction rate was calculated by measuring the BRET. Bioluminescence spectral scan measurements were performed at 37 °C using a Tecan SPARK multimode microplate reader after the addition of furimazine (Promega) at a dilution of 1:200 as previously described (49, 51, 52). BRET was calculated from the bioluminescence scan as indicated previously. The rate of reaction was calculated from the BRET using the equation below (16):

Rate={(BRETNoProtease−BRETProteaseBRETNoProtease)×[biosensor]}time

where BRET_No protease_ and BRET_Protease_ represent the BRET measured in the absence and presence of protease, respectively; [biosensor] is the DuProSense biosensor concentration, and time is the incubation period.

### Data analysis and figure preparation

GraphPad Prism (version 9, GraphPad Software, La Jolla, California, USA; www.graphpad.com), in combination with Microsoft Excel was used for data analysis and graph preparation. Some of the graphs were generated using Matplotlib (version 3.11), and python scripts for these were generated with the help of ChatGPT (GPT 5.5) and were verified by the authors. Figures were assembled using the Inkscape desktop application.

## Data availability

All relevant data of this study are available within this manuscript and the Supporting Information file.

## Supporting information

This article contains supporting information.

## Conflict of interest

The authors declare that they have no conflicts of interest with the contents of this article.
