# Supplementary material for: A P3′ methionine-to-phenylalanine substitution in the NSP6–7 substrate peptide alters SARS-CoV-2 Mpro dynamics and enhances its processing in living cells
Source: J Biol Chem. 2026 Aug 26;302(10):113495. doi: 10.1016/j.jbc.2026.113495 (PMC13661212; doi:10.1016/j.jbc.2026.113495)
Supplement: Supplementary file 1 [file mmc1.pdf]

Supporting Information

A P3' Methionine-to-Phenylalanine Substitution in the NSP6–7 Substrate Peptide Alters SARS-CoV-2 M^{pro} Dynamics and Enhances its Processing in Living Cells

Asma Fatima[1], Anupriya M Geethakumari[1,], Asfia Sultana[1], S M Nasir Uddin[1], Zeyaul Islam[2], Prasanna R Kolatkar[2], Kabir H Biswas[1,*]

Affiliations:
[1]College of Health & Life Sciences, Hamad Bin Khalifa University, Doha, Qatar
[2]Diabetes Research Center, Qatar Biomedical Research Institute, Hamad Bin Khalifa University, Doha, Qatar

*__Correspondence__: kbiswas@hbku.edu.qa

Supporting Text

Amino acid sequence of BRET-based SARS-CoV-2 DuProSense biosensors containing NSP6-7 WT and mutant cleavage site peptides

mNG-NSP4-5-NLuc-NSP6-7(K6G)- mSca:

MGSS**HHHHHH**SSGLVPRGSHMVSKGEEDNMASLPATHELHIFGSINGVDFDMVGQGTGNPNDGYEELNLKSTKGDLQFSPWILVPHIGYGFHQYLPY
PDGMSPFQAAMVDGSGYQVHRTMQFEDGASLTVNYRYTYEGSHIKGEAQVKGTGFPADGPVMTNSLTAADWCRSKKTYPNDKTIISTFKWSYTTGNG
KRYRSTARTTYTFAKPMAANYLKNQPMYVFRKTELKHSKTELNFKEWQKAFTDVMGMDELYKAAATENLYAVLQSGFRGSGSAMVFTLEDFVGDWRQ
TAGYNLDQVLEQGGVSSLFQNLGVSVTPIQRIVLSGENGLKIDIHVIIPYEGLSGDQMGQIEKIFKVVYPVDDHHFKVILHYGTLVIDGVTPNMIDY
FGRPYEGIAVFDGKKITVTGTLWNGNKIIDERLINPDGSLLFRVTINGVTGWRLCERILAGPRSGSATVQSGMSASQEFMVSKGEAVIKEFMRFKVH
MEGSMNGHEFEIEGEGEGRPYEGTQTAKLKVTKGGPLPFSWDILSPQFMYGSRAFTKHPADIPDYYKQSFPEGFKWERVMNFEDGGAVTVTQDTSLE
DGTLIYKVKLRGTNFPPDGPVMQKKTMGWEASTERLYPEDGVLKGDIKMALRLKDGGRYLADFKTTYKAKKPVQMPGAYNVDRKLDITSHNEDYTVV
EQYERSEGRHSTGGMDELYKGT**DYKDHDGDYKDHDIDYKDDDDKD**I

Highlights:
Bold: His-tag
Green: mNG
Yellow: NSP4-5 cleavage site
Blue: NLuc
Grey: NSP6-7 cleavage site
Red: mSca
Bold: 3×FLAG-tag
Theoretical pI: 5.89
Theoretical Mw: 81.3 kDa

mNG-NSP4-5-NLuc-NSP6-7(M7F)- mSca:

MGSS**HHHHHH**SSGLVPRGSHMVSKGEEDNMASLPATHELHIFGSINGVDFDMVGQGTGNPNDGYEELNLKSTKGDLQFSPWILVPHIGYGFHQYLPY
PDGMSPFQAAMVDGSGYQVHRTMQFEDGASLTVNYRYTYEGSHIKGEAQVKGTGFPADGPVMTNSLTAADWCRSKKTYPNDKTIISTFKWSYTTGNG
KRYRSTARTTYTFAKPMAANYLKNQPMYVFRKTELKHSKTELNFKEWQKAFTDVMGMDELYKAAATENLYAVLQSGFRGSGSAMVFTLEDFVGDWRQ
TAGYNLDQVLEQGGVSSLFQNLGVSVTPIQRIVLSGENGLKIDIHVIIPYEGLSGDQMGQIEKIFKVVYPVDDHHFKVILHYGTLVIDGVTPNMIDY
FGRPYEGIAVFDGKKITVTGTLWNGNKIIDERLINPDGSLLFRVTINGVTGWRLCERILAGPRSGSATVQSKFSASQEFMVSKGEAVIKEFMRFKVH
MEGSMNGHEFEIEGEGEGRPYEGTQTAKLKVTKGGPLPFSWDILSPQFMYGSRAFTKHPADIPDYYKQSFPEGFKWERVMNFEDGGAVTVTQDTSLE
DGTLIYKVKLRGTNFPPDGPVMQKKTMGWEASTERLYPEDGVLKGDIKMALRLKDGGRYLADFKTTYKAKKPVQMPGAYNVDRKLDITSHNEDYTVV
EQYERSEGRHSTGGMDELYKGT**DYKDHDGDYKDHDIDYKDDDDKD**I

Highlights:
Bold: His-tag
Green: mNG
Yellow: NSP4-5 cleavage site
Blue: NLuc
Grey: NSP6-7 cleavage site
Red: mSca
Bold: 3×FLAG-tag
Theoretical pI: 5.94
Theoretical Mw: 81.3 kDa

mNG-NSP4-5-NLuc-NSP6-7(S8R)- mSca:

MGSS**HHHHHH**SSGLVPRGSHMVSKGEEDNMASLPATHELHIFGSINGVDFDMVGQGTGNPNDGYEELNLKSTKGDLQFSPWILVPHIGYGFHQYLPY
PDGMSPFQAAMVDGSGYQVHRTMQFEDGASLTVNYRYTYEGSHIKGEAQVKGTGFPADGPVMTNSLTAADWCRSKKTYPNDKTIISTFKWSYTTGNG
KRYRSTARTTYTFAKPMAANYLKNQPMYVFRKTELKHSKTELNFKEWQKAFTDVMGMDELYKAAATENLYAVLQSGFRGSGSAMVFTLEDFVGDWRQ
TAGYNLDQVLEQGGVSSLFQNLGVSVTPIQRIVLSGENGLKIDIHVIIPYEGLSGDQMGQIEKIFKVVYPVDDHHFKVILHYGTLVIDGVTPNMIDY
FGRPYEGIAVFDGKKITVTGTLWNGNKIIDERLINPDGSLLFRVTINGVTGWRLCERILAGPRSGSATVQSKMRASQEFMVSKGEAVIKEFMRFKVH
MEGSMNGHEFEIEGEGEGRPYEGTQTAKLKVTKGGPLPFSWDILSPQFMYGSRAFTKHPADIPDYYKQSFPEGFKWERVMNFEDGGAVTVTQDTSLE
DGTLIYKVKLRGTNFPPDGPVMQKKTMGWEASTERLYPEDGVLKGDIKMALRLKDGGRYLADFKTTYKAKKPVQMPGAYNVDRKLDITSHNEDYTVV
EQYERSEGRHSTGGMDELYKGT**DYKDHDGDYKDHDIDYKDDDDKD**I

Highlights:
Bold: His-tag
Green: mNG
Yellow: NSP4-5 cleavage site

Blue: NLuc
Grey: NSP6-7 cleavage site
Red: mSca
Bold: 3×FLAG-tag
Theoretical pI: 5.99
Theoretical Mw: 81.4 kDa

Supporting Tables

Supporting Table 1. Table showing DOPE scores[52] of modeled protein structures generated by MODELLER (version 10.4)[48]. Models with the lowest DOPE scores for each M^{pro}–substrate peptide complex, highlighted in bold, were selected for subsequent analyses and MD simulations.

Structural Models	DOPE scores		
	Model 01	Model 02	Model 03
NSP4-5	**-77776.3**	-76869.4	-77458.6
NSP6-7	-77137.7	**-77237.2**	-77206.3
NSP6-7(M7F)	**-77558.5**	-76759.4	-76834.3

Supporting Table 2. Table showing the pLDDT values AlphaFold-derived M^{pro}-substrate peptide complexes.

Structural Models	pLDDT	
	Mpro	Sub. Pep
NSP4-5	91.54	53.82
NSP6-7	90.92	36.65
NSP6-7(M7F)	84.87	22.7

Supporting Table 3. Table showing cleavage kinetics parameters of M^{pro} determined from the live cells expressing NSP6-7 WT and mutant peptide DuProSense biosensors.

			$BRET_{max}$		$t_{1/2}$		Rate	
#	Protease	DuProSense biosensor	Green biosensor (%)(mean ± SD; *N* = 3)	Red biosensor (%)(mean ± SD; *N* = 3)	Green biosensor (h) (mean ± SD; *N* = 3)	Red biosensor (h)(mean ± SD; *N* = 3)	Green biosensor (h^{-1})(mean ± SD; *N* = 3)	Red biosensor (h^{-1}) (mean ± SD; *N* = 3)
1	M^{pro}	NSP4-5/6-7	101.36 ± 6.35	61.74 ± 9.54	8.52 ± 2.01	16.67 ± 3.49	0.23 ± 0.03	0.15 ± 0.03
2		NSP4-5/6-7(K6G)	90.81 ± 2.08	62.03 ± 2.51	9.62 ± 1.15	21.72 ± 1.33	0.51 ± 0.28	0.14 ± 0.03
3		NSP4-5/6-7 (M7F)	100.53 ± 5.52	85.14 ± 1	10.27 ± 1.12	22.46 ± 1.09	0.23 ± 0.02	0.22 ± 0.02
4		NSP4-5/6-7(S8R)	108.93 ± 10.44	68.64 ± 2.97	9.2 ± 1.35	22.55 ± 2.27	0.24 ± 0.07	0.22 ± 0.04

Supporting Table 4. Kinetic parameters of the M^{pro} obtained from the in vitro assay using purified DuProSense biosensors containing NSP6-7 WT and mutant peptide.

			V_{max} (fmol/s)			K_m (µM)		
#	Protease	DuProSense biosensor	Green biosensor (mean ± SD; *N* = 3)	Red biosensor (mean ± SD; *N* = 3)	p-value (Student's t-test) from red biosensor modules	Green biosensor (mean ± SD; *N* = 3)	Red biosensor (mean ± SD; *N* = 3)	p-value (Student's t-test) from red biosensor modules
1	M^{pro}	NSP4-5/6-7	9.77 ± 4.28	15.47 ± 3.93	0.165	1.03 ± 0.71	9.24 ± 3.58	0.018
2		NSP4-5/6-7(K6G)	8.69 ± 1.79	12.53 ± 10.11	0.553	0.76 ± 0.28	5.31 ± 3.99	0.028
3		NSP4-5/6-7 (M7F)	14.9 ± 0.65	2.91 ± 1.22	0.0001	2.54 ± 0.62	0.77 ± 0.66	0.120
4		NSP4-5/6-7(S8R)	5.94 ± 2.23	12.43 ± 9.95	0.332	0.43 ± 0.23	5.25 ± 3.91	0.100

Supporting Table 5. Table showing kinetic parameters of the NSP 6-7 WT and its mutant peptides obtained from the in vitro assay using purified DuProSense biosensors containing NSP6-7 WT and mutant peptide.

			V_{max} (fmol/s)		K_m (μM)		k_{cat}/K_m (s^{-1})	
#	Protease	DuProSense biosensor	Red biosensor (mean ± SD; *N* = 3)	p-value (Student's t-test) from red biosensor modules	Red biosensor (mean ± SD; *N* = 3)	p-value (Student's t-test) from red biosensor modules	Red biosensor (mean ± SD; *N* = 3)	p-value (Student's t-test) from red biosensor modules
1	M^{pro}	NSP4-5/6-7	15.47 ± 3.93		9.24 ± 3.58		0.009 ± 0.001	
2		NSP4-5/6-7(K6G)	12.53 ± 10.11	0.663	5.31 ± 3.99	0.273	0.13 ± 0.147	0.224
3		NSP4-5/6-7 (M7F)	2.91 ± 1.22	0.006	0.77 ± 0.66	0.016	0.004 ± 0.002	0.043
4		NSP4-5/6-7(S8R)	12.43 ± 9.95	0.648	5.25 ± 3.91	0.263	0.012 ± 0.001	0.030

Supporting Table 6. Table showing RMSF values of NSP4-5 and NSP6-7 substrate peptide residues bound to M^{pro}. RMSF values were calculated from 250 ns MD trajectories and compared using Student's t-test. Statistical analysis was performed using six independent monomer–substrate datasets (n = 6), obtained from the two M^{pro} monomers in each of three independent simulations. Data are presented as mean ± SD.

Residue	NSP4-5	NSP6-7	*p* -value
P4	1.13 ± 0.09	1.39 ± 0.12	0.0018
P3	1.04 ± 0.17	1.21 ± 0.11	0.0852
P2	1.18 ± 0.20	1.07 ± 0.11	0.3004
P1	0.81 ± 0.11	0.93 ± 0.15	0.1432
P1'	0.80 ± 0.07	1.04 ± 0.18	0.0193
P2'	0.97 ± 0.09	1.67 ± 0.13	0.0000
P3'	1.29 ± 0.20	2.19 ± 0.31	0.0002
P4'	1.63 ± 0.33	3.50 ± 0.58	0.0002

Supporting Table 7. Table showing RMSF values of NSP4-5 and NSP6-7(M7F) substrate peptide residues bound to Mpro. RMSF values were calculated from 250 ns MD trajectories and compared using Student's t-test. Statistical analysis was performed using six independent monomer–substrate datasets (n = 6), obtained from the two M^{pro} monomers in each of three independent simulations. Data are presented as mean ± SD.

Residue	NSP4-5	NSP6-7(M7F)	*p* -value
P4	1.13 ± 0.09	3.22 ± 4.06	0.2638
P3	1.04 ± 0.17	2.88 ± 3.60	0.2683
P2	1.18 ± 0.20	2.71 ± 3.76	0.3646
P1	0.81 ± 0.11	2.35 ± 3.45	0.3254
P1'	0.80 ± 0.07	2.39 ± 3.47	0.3128
P2'	0.97 ± 0.09	2.63 ± 3.12	0.2504
P3'	1.29 ± 0.20	2.97 ± 3.20	0.2549
P4'	1.63 ± 0.33	3.61 ± 2.94	0.1604

Supporting Table 8. Table showing RMSF values of NSP6-7 and NSP6-7(M7F) substrate peptide residues bound to Mpro. RMSF values were calculated from 250 ns MD trajectories and compared using Student's t-test. Statistical analysis was performed using six independent monomer–substrate datasets (n = 6), obtained from the two M^{pro} monomers in each of three independent simulations. Data are presented as mean ± SD.

Residue	NSP6-7	NSP6-7(M7F)	*p* -value
P4	1.39 ± 0.12	3.22 ± 4.06	0.3197
P3	1.21 ± 0.11	2.88 ± 3.60	0.3075
P2	1.07 ± 0.11	2.71 ± 3.76	0.3353
P1	0.93 ± 0.15	2.35 ± 3.45	0.3613
P1'	1.04 ± 0.18	2.39 ± 3.47	0.3851
P2'	1.67 ± 0.13	2.63 ± 3.12	0.4869
P3'	2.19 ± 0.31	2.97 ± 3.20	0.5812
P4'	3.50 ± 0.58	3.61 ± 2.94	0.9356

Supporting Figures

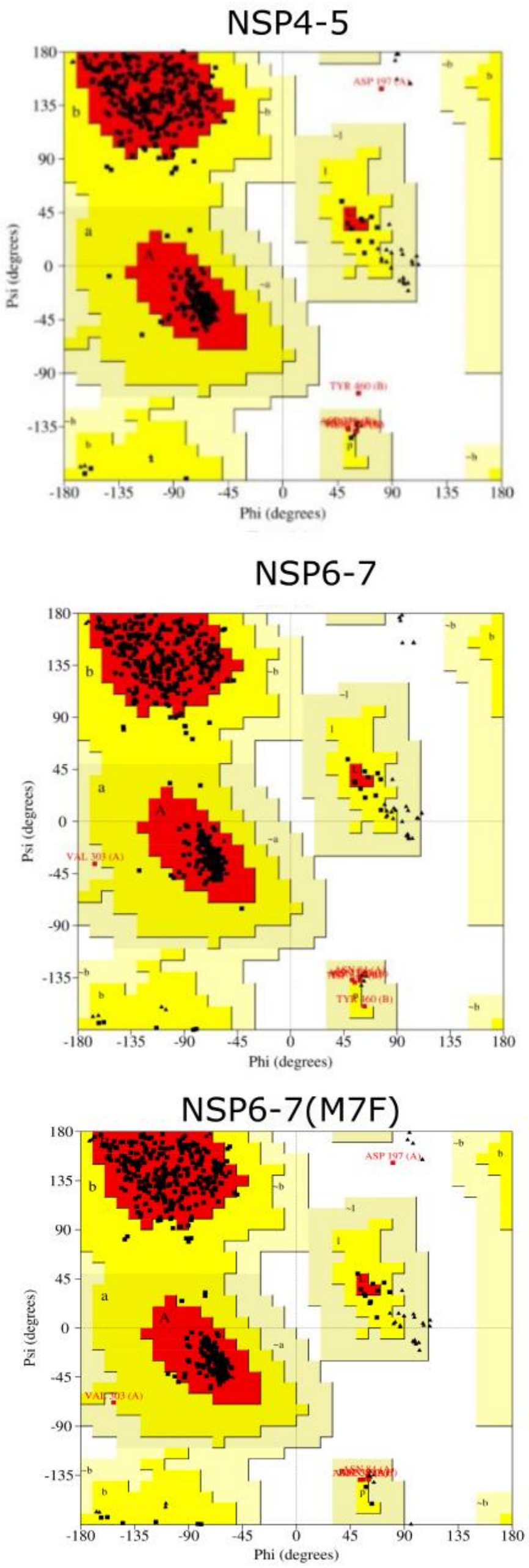

Supporting Figure 1.

Ramachandran plot analysis of dimeric M^pro structural models in complex with NSP4-5, NSP6-7, and NSP6-7(M7F) substrate peptides generated by MODELLER.

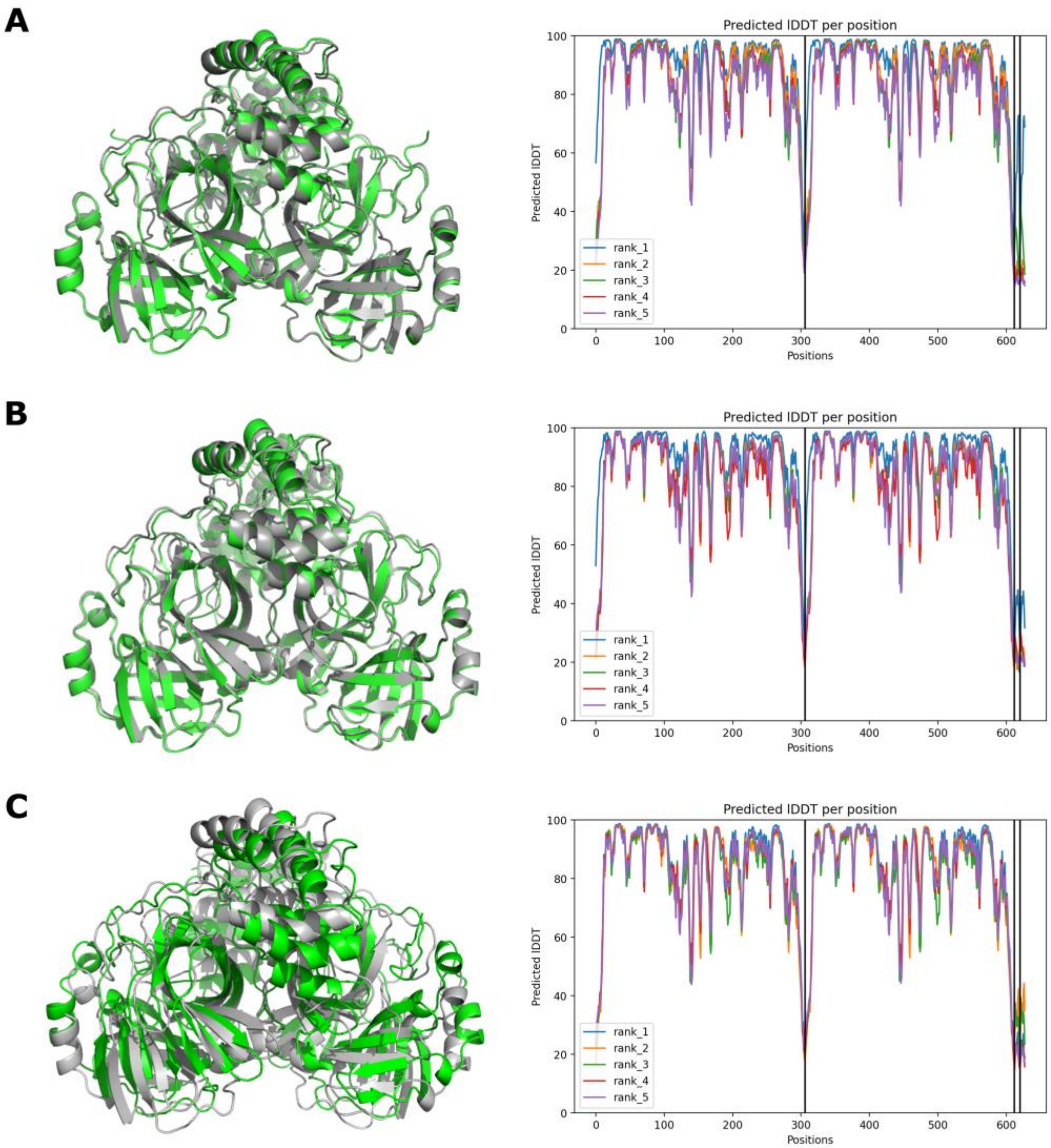

Supporting Figure 2.

Structural comparison and confidence assessment of Mpro–substrate peptide complexes predicted by AlphaFold2 and MODELLER. (A) NSP4-5, (B) NSP6-7, and (C) NSP6-7 (M7F) substrate complexes. Left panels show the structural alignment of M^{pro}–substrate models generated using AlphaFold2 (green) and MODELLER (gray). Models were aligned in PyMOL to evaluate structural agreement between the two modelling approaches. Right panels show the corresponding per-residue predicted Local Distance Difference Test (pLDDT) scores from AlphaFold2. The M^{pro} homodimer comprises residues 1–612, while residues 613-620 and 621–628 correspond to the substrate peptides bound to each monomer. High pLDDT values across the protein and substrate regions indicate high confidence in the predicted complex structures.

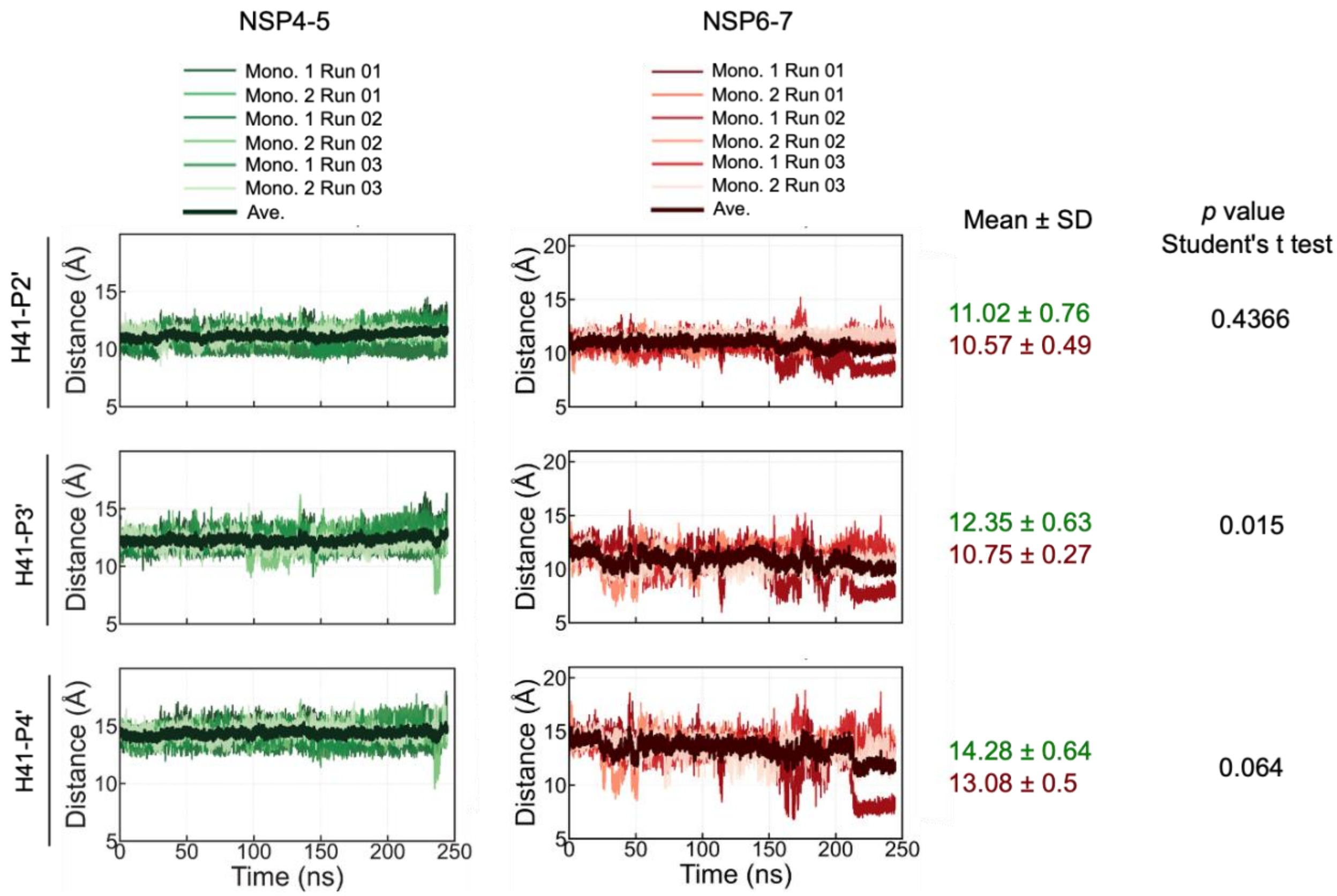

Supporting Figure 3.

Distance analysis of the C-terminal residues (P2’-P3’) of substrate peptide (NSP4-5 and NSP6-7) with the residue H41 of M^{pro}. Graphs showing the Cα–Cα distances between the indicated C-terminal residues (P2′ and P3′) of the NSP4-5 and NSP6-7 substrate peptides and the catalytic site residue H41 of M^{pro}, obtained from 250 ns all-atom explicit solvent MD simulations. The left panels display the distance variation over the simulation time for the indicated substrate peptides. The right panels show the corresponding distance values as mean ± SD from three independent MD simulations of the dimeric M^{pro}. Green and red colors represent NSP4-5 and NSP6-7 substrate peptides, respectively. Statistical significance was determined using an unpaired, two-tailed Student’s t-test, and *p*-values are indicated.

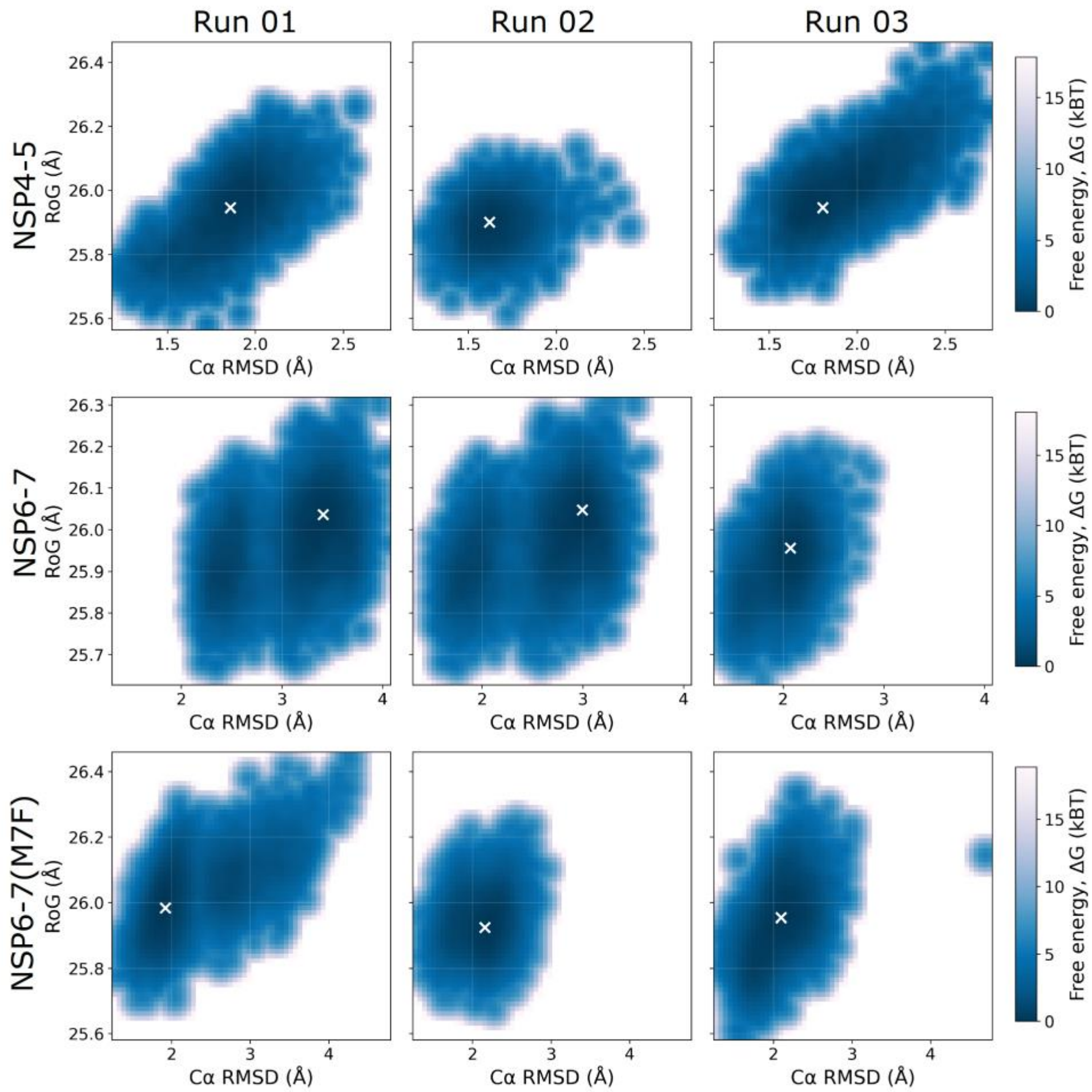

Supporting Figure 4.
Two-dimensional free-energy landscapes of M^{pro}-substrate peptide complexes across replicate simulations. Free-energy surfaces were constructed using RMSD and radius of gyration (RoG) as collective variables. Each replicate simulation was initiated using independent initial velocity seeds, enabling statistically independent trajectories.

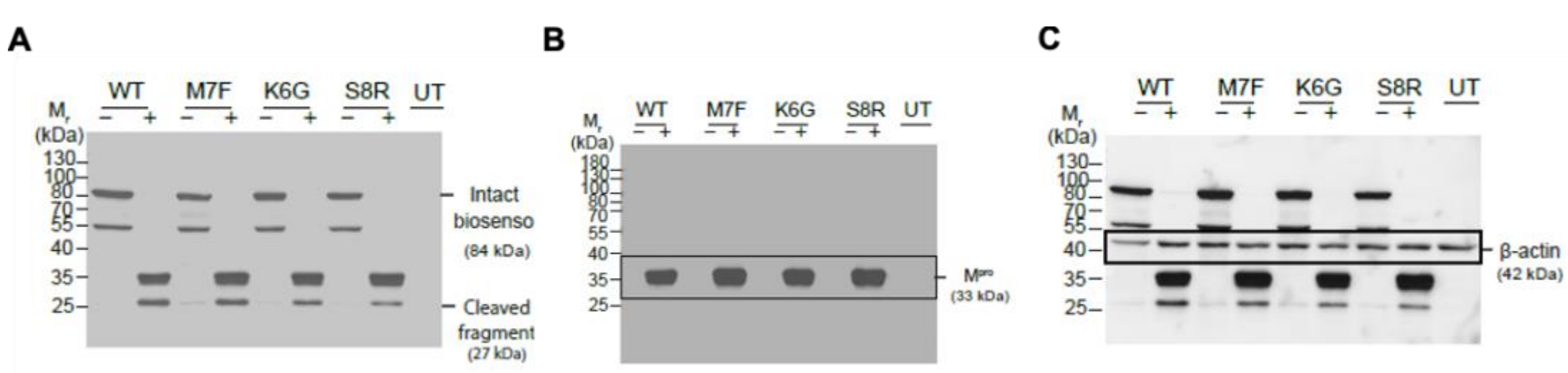

Supporting Figure 5.

(A-C) Full blots showing cleavage of biosensors containing NSP6-7 WT and mutant peptides probed with an anti-mNG antibody to detect cleaved fragments (~27 kDa) (A), an anti-M^{pro} antibody to confirm M^{pro} expression (B), and an anti-β-actin antibody (loading control) (C).

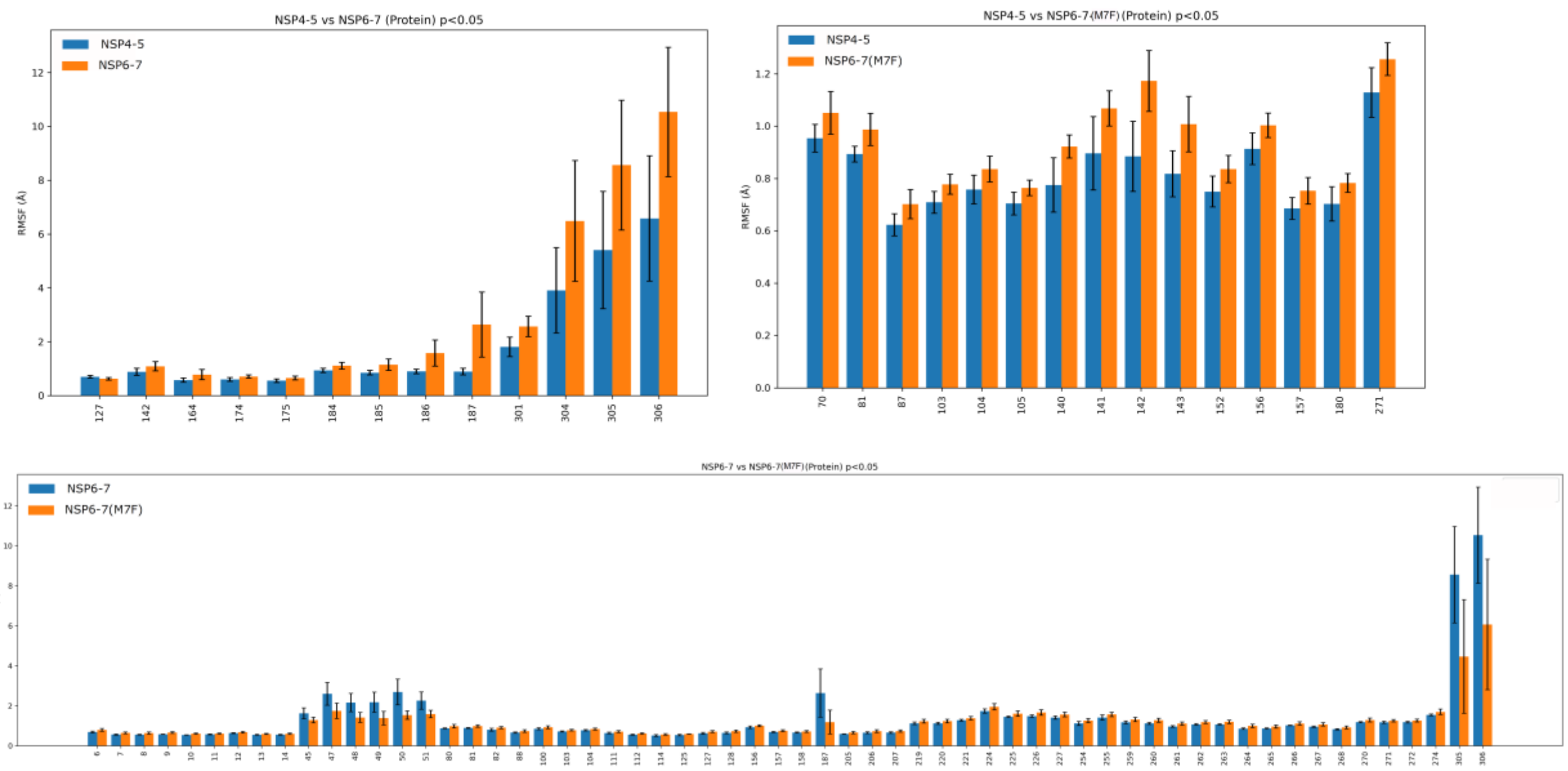

Supporting Figure 6. Residue-wise RMSF comparison of substrate peptides and M^{pro} across different systems. NSP4–5 vs NSP6–7 (left), NSP4–5 vs NSP6–7(M7F) (top right), and NSP6–7 vs NSP6–7(M7F) (bottom). Data represent average values obtained from each monomer across three independent 250 ns MD simulations. Error bars indicate standard deviation (SD). Only statistically significant differences (p < 0.05), determined using Student's t-test, are shown.

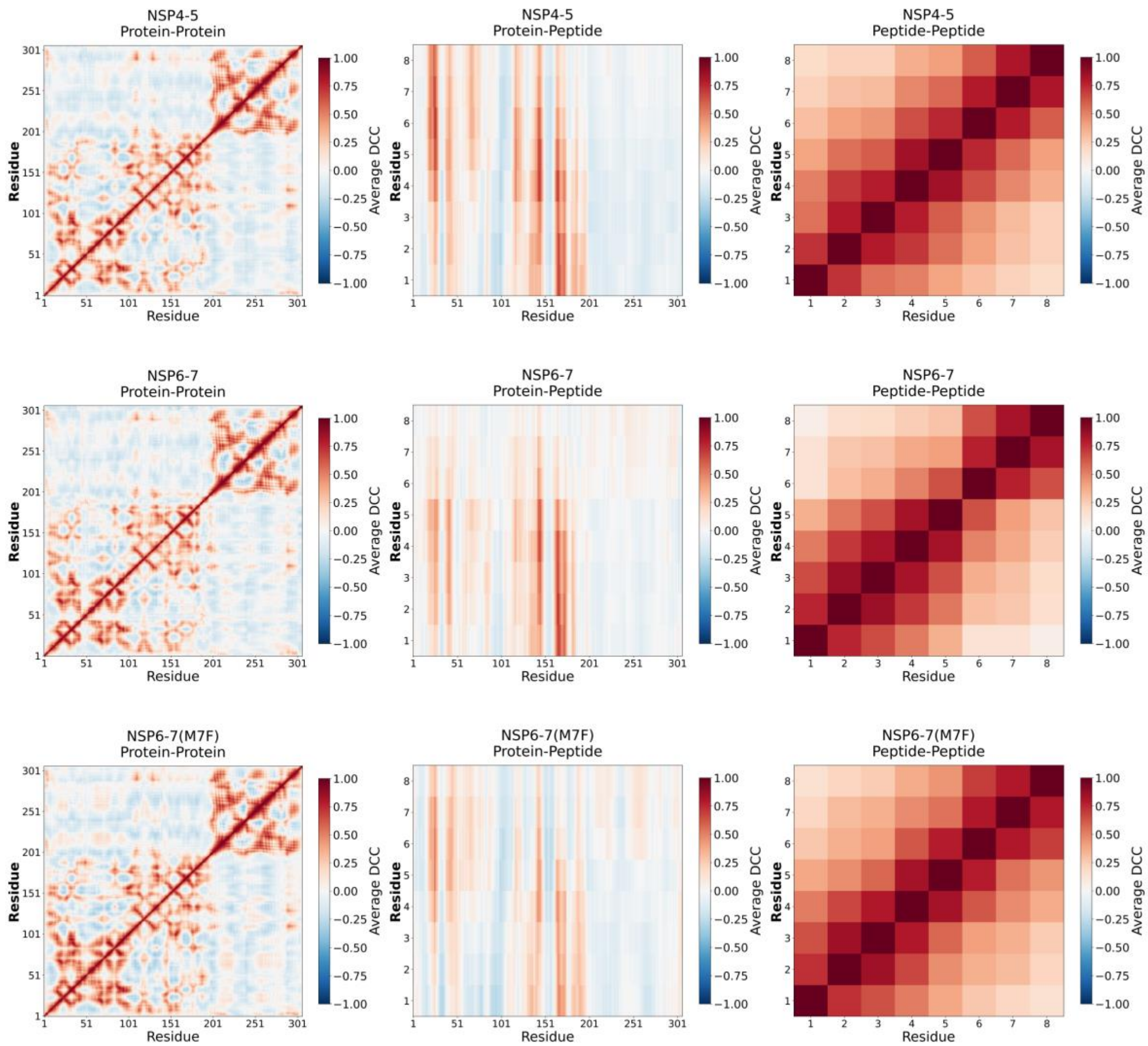

Supporting Figure 7. Dynamic cross-correlation analysis of average residue motions within M^{pro} (left), between M^{pro} and the substrate peptide (middle), and within the substrate peptide (right).
Data shown are obtained from each monomer across three independent 250 ns-long MD simulations. *p*-values were obtained using Student's t-test analysis.

References

1 Lee, J. *et al.* X-ray crystallographic characterization of the SARS-CoV-2 main protease polyprotein cleavage sites essential for viral processing and maturation. *Nature Communications* **13**, 5196, doi:10.1038/s41467-022-32854-4 (2022).

2 Owen, D. R. *et al.* An oral SARS-CoV-2 M(pro) inhibitor clinical candidate for the treatment of COVID-19. *Science* **374**, 1586-1593, doi:10.1126/science.abl4784 (2021).

3 Abdelnabi, R. *et al.* The oral protease inhibitor (PF-07321332) protects Syrian hamsters against infection with SARS-CoV-2 variants of concern. *Nat Commun* **13**, 719, doi:10.1038/s41467-022-28354-0 (2022).

4 Reina, J. & Iglesias, C. [Nirmatrelvir plus ritonavir (Paxlovid) a potent SARS-CoV-2 3CLpro protease inhibitor combination]. *Rev Esp Quimioter* **35**, 236-240, doi:10.37201/req/002.2022 (2022).

5 Shin, D. *et al.* Papain-like protease regulates SARS-CoV-2 viral spread and innate immunity. *Nature* **587**, 657-662, doi:10.1038/s41586-020-2601-5 (2020).

6 Ferreira, J. C., Fadl, S. & Rabeh, W. M. Key dimer interface residues impact the catalytic activity of 3CLpro, the main protease of SARS-CoV-2. *J. Biol. Chem.* **298**, 102023, doi:10.1016/j.jbc.2022.102023 (2022).

7 Narwal, M., Armache, J. P., Edwards, T. J. & Murakami, K. S. SARS-CoV-2 polyprotein substrate regulates the stepwise M(pro) cleavage reaction. *J. Biol. Chem.* **299**, 104697, doi:10.1016/j.jbc.2023.104697 (2023).

8 Fatima, A., Geethakumari, A. M., Uddin, S. M. & Biswas, K. H. Differential Kinetics of SARS-CoV-2 Proteases Revealed by a Dual-Color, BRET-based Protease Biosensor, DuProSense. *bioRxiv*, 2024.2009.2026.615113, doi:10.1101/2024.09.26.615113 (2024).

9 Krichel, B., Falke, S., Hilgenfeld, R., Redecke, L. & Uetrecht, C. Processing of the SARS-CoV pp1a/ab nsp7-10 region. *Biochem J* **477**, 1009-1019, doi:10.1042/BCJ20200029 (2020).

10 MacDonald, E. A. *et al.* Recognition of Divergent Viral Substrates by the SARS-CoV-2 Main Protease. *ACS Infect Dis* **7**, 2591-2595, doi:10.1021/acsinfecdis.1c00237 (2021).

11 Schamoni-Kast, K. *et al.* The kinetics of SARS-CoV-2 nsp7-11 polyprotein processing and impact on complexation with nsp16. *bioRxiv*, 2024.2001.2006.574466, doi:10.1101/2024.01.06.574466 (2024).

12 Yaghi, R. M., Andrews, C. L., Wylie, D. C. & Iverson, B. L. High-Resolution Substrate Specificity Profiling of SARS-CoV-2 M(pro); Comparison to SARS-CoV M(pro). *ACS Chem Biol* **19**, 1474-1483, doi:10.1021/acschembio.4c00096 (2024).

13 Kenward, C., Vuckovic, M., Paetzel, M. & Strynadka, N. C. J. Kinetic comparison of all eleven viral polyprotein cleavage site processing events by SARS-CoV-2 main protease using a linked protein FRET platform. *J. Biol. Chem.* **300**, 107367, doi:https://doi.org/10.1016/j.jbc.2024.107367 (2024).

14 Yadav, R. *et al.* Biochemical and structural insights into SARS-CoV-2 polyprotein processing by Mpro. *Science Advances* **8**, eadd2191, doi:doi:10.1126/sciadv.add2191 (2022).

15 Zhao, Y. *et al.* Structural basis for replicase polyprotein cleavage and substrate specificity of main protease from SARS-CoV-2. *Proc. Natl. Acad. Sci. U. S. A.* **119**, e2117142119, doi:10.1073/pnas.2117142119 (2022).

16 Lee, J. *et al.* X-ray crystallographic characterization of the SARS-CoV-2 main protease polyprotein cleavage sites essential for viral processing and maturation. *Nat. Commun.* **13**, 5196, doi:10.1038/s41467-022-32854-4 (2022).

17 Rut, W. *et al.* SARS-CoV-2 M(pro) inhibitors and activity-based probes for patient-sample imaging. *Nat. Chem. Biol.* **17**, 222-228, doi:10.1038/s41589-020-00689-z (2021).

18 Jin, Z. *et al.* Structure of Mpro from SARS-CoV-2 and discovery of its inhibitors. *Nature* **582**, 289-293, doi:10.1038/s41586-020-2223-y (2020).

19 Zhao, Y. *et al.* Structural basis for replicase polyprotein cleavage and substrate specificity of main protease from SARS-CoV-2. *Proceedings of the National Academy of Sciences* **119**, e2117142119, doi:doi:10.1073/pnas.2117142119 (2022).

20 El-Maradny, Y. A. *et al.* Unraveling the role of the nucleocapsid protein in SARS-CoV-2 pathogenesis: From viral life cycle to vaccine development. *International Journal of Biological Macromolecules* **279**, 135201, doi:https://doi.org/10.1016/j.ijbiomac.2024.135201 (2024).

21 Thoms, M. *et al.* Structural basis for translational shutdown and immune evasion by the Nsp1 protein of SARS-CoV-2. *Science* **369**, 1249-1255 (2020).

22 Chan, H. T. H., Oliveira, A. S. F., Mulholland, A. J., Schofield, C. J. & Duarte, F. Substrate recognition and selectivity in SARS-CoV-2 main protease: Unveiling the role of subsite interactions through dynamical nonequilibrium molecular dynamics simulations. *bioRxiv*, 2023.2012.2001.569046, doi:10.1101/2023.12.01.569046 (2023).
23 Ricciardi, S. *et al.* The role of NSP6 in the biogenesis of the SARS-CoV-2 replication organelle. *Nature* **606**, 761-768, doi:10.1038/s41586-022-04835-6 (2022).
24 Zhao, M. *et al.* SARS-CoV-2 polyprotein expression and the induction of double-membrane vesicles. *J. Virol.* **99**, e0138525, doi:10.1128/jvi.01385-25 (2025).
25 Lee, J. G. *et al.* Characterization of SARS-CoV-2 proteins reveals Orf6 pathogenicity, subcellular localization, host interactions and attenuation by Selinexor. *Cell Biosci* **11**, 58, doi:10.1186/s13578-021-00568-7 (2021).
26 Bhardwaj, T. *et al.* Amyloidogenic proteins in the SARS-CoV and SARS-CoV-2 proteomes. *Nature Communications* **14**, 945, doi:10.1038/s41467-023-36234-4 (2023).
27 te Velthuis, A. J. W., van den Worm, S. H. E. & Snijder, E. J. The SARS-coronavirus nsp7+nsp8 complex is a unique multimeric RNA polymerase capable of both de novo initiation and primer extension. *Nucleic Acids Research* **40**, 1737-1747, doi:10.1093/nar/gkr893 (2011).
28 Singh, D. *et al.* Redefining NSP12 activity in SARS-CoV-2 and its regulation by NSP8 and NSP7. *Molecular Therapy Nucleic Acids* **36**, 102452, doi:https://doi.org/10.1016/j.omtn.2025.102452 (2025).
29 Fiser, A. & Sali, A. Modeller: generation and refinement of homology-based protein structure models. *Methods Enzymol* **374**, 461-491, doi:10.1016/s0076-6879(03)74020-8 (2003).
30 Phillips, J. C. *et al.* Scalable Molecular Dynamics With NAMD. *Journal of computational chemistry* **26**, 1781-1802 (2005).
31 Humphrey, W., Dalke, A. & Schulten, K. VMD: visual molecular dynamics. *J Mol Graph* **14**, 33-38, 27-38, doi:10.1016/0263-7855(96)00018-5 (1996).
32 WL, D. *The PyMOL molecular graphics system.*, < http://www.pymol.org> (2002).
33 Yang, K. S., Leeuwon, S. Z., Xu, S. & Liu, W. R. Evolutionary and Structural Insights about Potential SARS-CoV-2 Evasion of Nirmatrelvir. *J. Med. Chem.* **65**, 8686-8698, doi:10.1021/acs.jmedchem.2c00404 (2022).
34 Parigger, L. *et al.* Recent changes in the mutational dynamics of the SARS-CoV-2 main protease substantiate the danger of emerging resistance to antiviral drugs. *Front Med (Lausanne)* **9**, 1061142, doi:10.3389/fmed.2022.1061142 (2022).
35 Ramos-Guzmán, C. A., Ruiz-Pernía, J. J. & Tuñón, I. Unraveling the SARS-CoV-2 Main Protease Mechanism Using Multiscale Methods. *ACS Catalysis* **10**, 12544-12554, doi:10.1021/acscatal.0c03420 (2020).
36 Shaner, N. C. *et al.* A bright monomeric green fluorescent protein derived from Branchiostoma lanceolatum. *Nat Methods* **10**, 407-409, doi:10.1038/nmeth.2413 (2013).
37 Hall, M. P. *et al.* Engineered luciferase reporter from a deep sea shrimp utilizing a novel imidazopyrazinone substrate. *ACS Chem Biol* **7**, 1848-1857, doi:10.1021/cb3002478 (2012).
38 Bindels, D. S. *et al.* mScarlet: a bright monomeric red fluorescent protein for cellular imaging. *Nat Methods* **14**, 53-56, doi:10.1038/nmeth.4074 (2017).
39 Geethakumari, A. M. *et al.* A BRET-based Mpro biosensor containing a nanobody and tandem cleavage sites shows an increased cleavage rate. *Sensors and Actuators Reports* **9**, 100315, doi:https://doi.org/10.1016/j.snr.2025.100315 (2025).
40 Sultana, A., Geethakumari, A. M., Islam, Z., Kolatkar, P. R. & Biswas, K. H. BRET-based biosensors for SARS-CoV-2 oligonucleotide detection. *Front Bioeng Biotechnol* **12**, 1353479, doi:10.3389/fbioe.2024.1353479 (2024).
41 Ahmed, W. S. *et al.* Coevolving Residues Distant from the Ligand Binding Site are Involved in GAF Domain Function. *bioRxiv*, 2024.2008.2007.605472, doi:10.1101/2024.08.07.605472 (2024).
42 Ahmed, W. S. *et al.* A slow but steady nanoLuc: R162A mutation results in a decreased, but stable, nanoLuc activity. *Int. J. Biol. Macromol.*, 131864, doi:https://doi.org/10.1016/j.ijbiomac.2024.131864 (2024).
43 Geethakumari, A. M. *et al.* A genetically encoded BRET-based SARS-CoV-2 M(pro) protease activity sensor. *Commun Chem* **5**, 117, doi:10.1038/s42004-022-00731-2 (2022).
44 Altamash, T., Ahmed, W., Rasool, S. & Biswas, K. H. Intracellular Ionic Strength Sensing Using NanoLuc. *Int J Mol Sci* **22**, doi:10.3390/ijms22020677 (2021).

45 Huang, J. *et al.* CHARMM36m: an improved force field for folded and intrinsically disordered proteins. *Nature methods* **14**, 71-73 (2017).
46 Shaqra, A. M. *et al.* Defining the substrate envelope of SARS-CoV-2 main protease to predict and avoid drug resistance. *Nat Commun* **13**, 3556, doi:10.1038/s41467-022-31210-w (2022).
47 Phillips, J. C. *et al.* Scalable molecular dynamics on CPU and GPU architectures with NAMD. *J Chem Phys* **153**, 044130, doi:10.1063/5.0014475 (2020).
48 Brooks, B. R. *et al.* CHARMM: The biomolecular simulation program. *Journal of Computational Chemistry* **30**, 1545-1614, doi:https://doi.org/10.1002/jcc.21287 (2009).
49 Philip, A. M., Ahmed, W. S. & Biswas, K. H. Reversal of the unique Q493R mutation increases the affinity of Omicron S1-RBD for ACE2. *Computational and Structural Biotechnology Journal* **21**, 1966-1977, doi:https://doi.org/10.1016/j.csbj.2023.02.019 (2023).
50 Ahmed, W. S., Philip, A. M. & Biswas, K. H. Decreased Interfacial Dynamics Caused by the N501Y Mutation in the SARS-CoV-2 S1 Spike:ACE2 Complex. *Front Mol Biosci* **9**, 846996, doi:10.3389/fmolb.2022.846996 (2022).
51 Gordon, D. E. *et al.* A SARS-CoV-2 protein interaction map reveals targets for drug repurposing. *Nature* **583**, 459-468, doi:10.1038/s41586-020-2286-9 (2020).
52 Sultana, A., Geethakumari, A. M., Islam, Z., Kolatkar, P. R. & Biswas, K. H. BRET-based biosensors for SARS-CoV-2 oligonucleotide detection. *Frontiers in Bioengineering and Biotechnology* **12**, doi:10.3389/fbioe.2024.1353479 (2024).
53 Ahmed, W. S. *et al.* A slow but steady nanoLuc: R162A mutation results in a decreased, but stable, nanoLuc activity. *Int J Biol Macromol* **269**, 131864, doi:10.1016/j.ijbiomac.2024.131864 (2024).
54 Fatima, A., Geethakumari, A. M., Ahmed, W. S. & Biswas, K. H. A potential allosteric inhibitor of SARS-CoV-2 main protease (M(pro)) identified through metastable state analysis. *Front Mol Biosci* **11**, 1451280, doi:10.3389/fmolb.2024.1451280 (2024).
55 Jan, Z., Geethakumari, A. M., Biswas, K. H. & Jithesh, P. V. Protegrin-2, a potential inhibitor for targeting SARS-CoV-2 main protease M(pro). *Comput Struct Biotechnol J* **21**, 3665-3671, doi:10.1016/j.csbj.2023.07.020 (2023).
56 Geethakumari, A. M. *et al.* A genetically encoded BRET-based SARS-CoV-2 Mpro protease activity sensor. *Communications Chemistry* **5**, 117, doi:10.1038/s42004-022-00731-2 (2022).
57 Mihalič, F. *et al.* Identification of motif-based interactions between SARS-CoV-2 protein domains and human peptide ligands pinpoint antiviral targets. *Nature Communications* **14**, 5636, doi:10.1038/s41467-023-41312-8 (2023).
58 Fatima, A., Geethakumari, A. M., Ahmed, W. S. & Biswas, K. H. A potential allosteric inhibitor of SARS-CoV-2 main protease (Mpro) identified through metastable state analysis. *Frontiers in Molecular Biosciences* **11**, doi:10.3389/fmolb.2024.1451280 (2024).
